# Multimerin 1 supports platelet function in vivo and binds to specific GPAGPOGPX motifs in fibrillar collagens that enhance platelet adhesion

**DOI:** 10.1111/jth.15171

**Published:** 2020-12-17

**Authors:** Alexander Leatherdale, D’Andra Parker, Subia Tasneem, Yiming Wang, Dominique Bihan, Arkadiusz Bonna, Samir W. Hamaia, Peter L. Gross, Heyu Ni, Bradley W. Doble, David Lillicrap, Richard W. Farndale, Catherine P. M. Hayward

**Affiliations:** ^1^ Pathology and Molecular Medicine McMaster University Hamilton ON Canada; ^2^ Laboratory Medicine and Pathobiology Keenan Research Centre Li Ka‐Shing Knowledge Institute St. Michael's Hospital University of Toronto Toronto ON Canada; ^3^ Canadian Blood Services Centre for Innovation Ottawa ON Canada; ^4^ Biochemistry, Downing Site University of Cambridge Cambridge UK; ^5^ Medicine, Thrombosis and Atherosclerosis Research Institute McMaster University Hamilton ON Canada; ^6^ Biochemistry and Biomedical Sciences McMaster Stem Cell and Cancer Research Institute McMaster University Hamilton ON Canada; ^7^ Pathology and Molecular Medicine Richardson Laboratory Queen’s University Kingston ON Canada; ^8^ Hamilton Regional Laboratory Medicine Program, and Department of Medicine McMaster University Hamilton ON Canada; ^9^ Present address: CambCol Laboratories Ltd Ely UK

**Keywords:** blood platelets, fibrillar collagens, multimerin, platelet adhesiveness, von Willebrand factor

## Abstract

**Background:**

Multimerin 1 (human: MMRN1, mouse: Mmrn1) is a homopolymeric, adhesive, platelet and endothelial protein that binds to von Willebrand factor and enhances platelet adhesion to fibrillar collagen ex vivo.

**Objectives:**

To examine the impact of Mmrn1 deficiency on platelet adhesive function, and the molecular motifs in fibrillar collagen that bind MMRN1 to enhance platelet adhesion.

**Methods:**

Mmrn1‐deficient mice were generated and assessed for altered platelet adhesive function. Collagen Toolkit peptides, and other triple‐helical collagen peptides, were used to identify multimerin 1 binding motifs and their contribution to platelet adhesion.

**Results:**

MMRN1 bound to conserved GPAGPOGPX sequences in collagens I, II, and III (including GPAGPOGPI, GPAGPOGPV, and GPAGPOGPQ) that enhanced activated human platelet adhesion to collagen synergistically with other triple‐helical collagen peptides (*P* < .05). *Mmrn1^−/−^* and *Mmrn1^+/−^* mice were viable and fertile, with complete and partial platelet Mmrn1 deficiency, respectively. Relative to wild‐type mice, *Mmrn1^−/−^* and *Mmrn1^+/−^* mice did not have overt bleeding, increased median bleeding times, or increased wound blood loss (*P* ≥ .07); however, they both showed significantly impaired platelet adhesion and thrombus formation in the ferric chloride injury model (*P* ≤ .0003). *Mmrn1^−/−^* platelets had impaired adhesion to GPAGPOGPX peptides and fibrillar collagen (*P* ≤ .03) and formed smaller aggregates than wild‐type platelets when captured onto collagen, triple‐helical collagen mimetic peptides, von Willebrand factor, or fibrinogen (*P* ≤ .008), despite preserved, low shear, and high shear aggregation responses.

**Conclusions:**

Multimerin 1 supports platelet adhesion and thrombus formation and binds to highly conserved, GPAGPOGPX motifs in fibrillar collagens that synergistically enhance platelet adhesion.

**Essentials**

- Multimerin 1 (Mmrn1) is a homopolymeric protein that supports platelet adhesion in vitro.
- Mmrn1‐deficient mice and collagen peptides were used to assess Mmrn1 contributions to platelet function.
- Mmrn1 loss impaired platelet adhesion in vivo and to GPAGPOGPX motifs in collagens in vitro.
- Mmrn1 contributes to platelet function and binds to adhesive GPAGPOGPX motifs in collagen.

## 1 INTRODUCTION

Platelet adhesion is a critical step in hemostasis and thrombosis that allows platelets to localize and accumulate at sites of vessel injury or thrombus formation. Multimerin 1 (human: MMRN1, mouse: Mmrn1) is a large, soluble, homopolymeric adhesive glycoprotein that is synthesized and stored by megakaryocytes/platelets and endothelial cells for regulated release, but is undetectable in normal plasma.^1^, ^2^, ^3^, ^4^ When released, MMRN1 supports platelet adhesion through shear‐dependent mechanisms involving activated α_IIb_β_3_ and α_V_β_3_ under static conditions and low shear flow conditions (≤150 s^−1^), and involving von Willebrand factor (human: VWF; mouse: Vwf) and GPIbα, but not β_3_ integrins, under high shear flow (1500 s^−1^).^5^, ^6^ MMRN1 binds to VWF with high affinity through a two‐site, two‐step interaction with the VWF A1 and A3 domains that enhances platelet adhesion to immobilized VWF at high shear flow.^7^ MMRN1 also enhances platelet adhesion to vascular fibrillar collagens I and III and Horm collagen (equine collagen I, ~95%, and III, ~5%) under high shear flow through mechanisms requiring VWF and GPIbα^6^ and uncharacterized motifs in collagen that support MMRN1 binding. The impact of Mmrn1 deficiency on platelet function has not been fully characterized.

Similar to MMRN1, the adhesive proteins VWF, fibronectin (FN), vitronectin (VN), and fibrin self‐associate to form large homopolymers,^8^, ^9^, ^10^, ^11^, ^12^, ^13^, ^14^ and bind to α_IIb_β_3_ on platelets^15^, ^16^, ^17^, ^18^, ^19^, ^20^ to mediate aggregate formation. Additionally, thrombospondin‐1 (TSP‐1) helps to crosslink platelets by self‐associating and binding α_IIb_β_3_‐bound fibrinogen (FG).^21^ These interactions create large macromolecular complexes that increase the likelihood of platelet–platelet collisions and the avidity of platelet–matrix or platelet–platelet interactions.^22^ We postulated that the large MMRN1 homopolymers released by platelets and endothelial cells might similarly enhance platelet–matrix or platelet–platelet interactions, as *Mmrn1^−/−^Snca^−/−^* mice (evaluated by the ferric chloride [FeCl_3_] mesenteric vessel injury model) have impaired platelet adhesion and thrombus formation in vivo, and impaired platelet adhesion to collagen in vitro, that are corrected by exogenous MMRN1.^23^

We generated Mmrn1‐deficient (*Mmrn1^−/−^*) mice to: (a) determine the impact of selective Mmrn1 loss on platelet adhesion and thrombus formation in vivo; (b) identify additional adhesive ligands for MMRN1/Mmrn1, and the impact of Mmrn1 deficiency on platelet adhesion and aggregation; and (c) investigate the motifs in collagen that support binding to MMRN1. We demonstrate that Mmrn1 contributes to platelet adhesion and thrombus formation in vivo and update the current model of platelet adhesion to collagen to include GPAGPOGPX, a conserved MMRN1/Mmrn1 binding motif that synergistically enhances platelet adhesion.

## 2 METHODS

The study was conducted in accordance with the recently revised Declaration of Helsinki with approval of the Hamilton Integrated Research Ethics Board, McMaster University Animal Research Ethics Board, and St. Michael's Animal Care Committee.

Mmrn1‐deficient mice were generated as outlined in Appendix S1 in supporting information. Experiments were done with wild‐type, *Mmrn1^−/−^*, and *Mmrn1^+/−^* mice obtained from crosses of *Mmrn1^+/−^* mice that were regenerated every three to five generations by additional crosses, as outlined in Appendix S1.

### 2.1 Evaluation of mouse bleeding after tail transection

Bleeding times (BT) and blood loss following tail transection 1.5 mm from the distal tip were evaluated as described.^24^ BT were recorded as 900 seconds if bleeding had not stopped by then.

### 2.2 Blood collection

Murine blood was obtained by terminal exsanguination of anesthetized mice after carotid artery cannulation.^25^ Human blood was collected from general population controls with written informed consent.

### 2.3 Mouse blood counts and glycoprotein analysis

Complete blood counts were determined using a Hemavet 950 instrument (Drew Scientific Inc). Platelet Mmrn1 content was evaluated by western blot analysis of platelet lysate after reduced sodium dodecyl sulfate polyacrylamide gel electrophoresis (SDS‐PAGE) using polyclonal rabbit antibodies (SC‐367225, Santa Cruz Biotechnology, Inc).^26^ Plasma and platelet Vwf were quantified by enzyme‐linked immunosorbent assay (ELISA) using polyclonal rabbit anti‐human VWF (A0082 and P0226, DAKO Canada Inc) and pooled normal mouse plasma (NMP) as the standard (results reported as % NMP) as described.^27^, ^28^ Flow cytometry was performed as described,^25^ using: fluorescein isothiocyanate (FITC)‐labeled rat anti‐mouse GpIbα (CD42b; clone Xia.G5), anti‐mouse/rat P‐selectin (CD62P; Emfret Analytics; clone Wug.E9) and rat anti‐mouse β_1_ (CD29; BD Biosciences; clone Ha2/5), and phycoerythrin (PE)‐labeled hamster anti‐mouse β_3_ (CD61, BD Biosciences; clone 2C9.G2) and rat anti‐mouse activated α_IIb_β_3_ (CD41/CD61, Emfret Analytics; clone JON/A).

### 2.4 Intravital microscopy

Platelet adhesion and thrombus formation in mesenteric arterioles treated with 250 mmol/L FeCl_3_ were evaluated as described,^23^ to assess: (a) fluorescent platelet deposition on the vessel wall per minute, between 3 and 5 minutes following injury, (b) time to form the first 20 µm diameter thrombus, and (c) vessel occlusion time.

### 2.5 Preparation of recombinant human multimerin 1

Recombinant (r) human MMRN1 was affinity purified from media of stably transfected human embryonic kidney (HEK)‐293 cells and assessed for concentration and purity by ELISA, western blotting, and silver staining as described.^4^, ^5^

### 2.6 Protein binding assays

rMMRN1 binding to immobilized human FG (±pre‐treatment with 0.2 U/mL thrombin to induce fibrin formation), FN, or triple‐helical collagen peptides was evaluated based on methods described,^6^ using Immulon 2 HB flat‐bottom plates (Thermo Fisher Scientific) coated (overnight, 4°C) with 1 µg/well protein or peptide.

### 2.7 Assays of platelet adhesion under shear

Endpoint and real‐time analyses of mouse platelet adhesion were evaluated as described,^7^ except Vena8Fluoro+ (Cellix Ltd) biochips were coated (overnight, 4°C) with: 100 µg/mL Horm collagen (Nycomed Austria GmbH), 10 U/mL recombinant (r)Vwf, 100 µg/mL murine fibrinogen (Fg; ±15 minutes pre‐treatment with 0.2 U/mL thrombin after immobilization to convert to fibrin, as described)^5^ (Molecular Innovations), 100 µg/mL murine fibronectin (Fn; Molecular Innovations), or combinations of triple‐helical collagen peptides (50 µg/mL of each, or 100 µg/mL GPP). For testing adhesion of *Mmrn1^−/−^* and wild‐type platelets in whole blood to Fg, fibrin, Fn, and rVwf, blood was collected into 93 µmol/L (50 µg/mL) PPACK, or into 93 µmol/L PPACK with 2 U/mL heparin for experiments with Horm collagen,^6^, ^23^ before labeling platelets with 4 µmol/L DiOC6(3) (Enzo Life Sciences; 10 minutes, 37°C, ≥5 × 10^8^ platelets/mL). *Mmrn1^−/−^* and wild‐type whole blood for real‐time perfusion assays was collected into 10 U/mL heparin.^23^ For assays with washed mouse platelets, samples were prepared and labeled with 4 µmol/L DiOC6(3) as described^7^ and incubated with 10 µg/mL crosslinked collagen‐related peptide (CRP‐XL; sequence: GCO[GPO_10_]GCOG, hereafter referred to as CRP) to induce Mmrn1 release before testing.

### 2.8 Mouse platelet aggregation

Light transmission aggregometry (LTA) and whole blood aggregometry (WBA) were performed using: *Mmrn1^−/−^* and wild‐type mouse samples; a Chrono‐Log aggregometer (Havertown, PA; settings as recommended by the manufacturer)^23^; mouse whole blood, platelet‐rich plasma (PRP), and gel‐filtered platelets (GFP, thrombin‐induced aggregation only), with PRP and GFP adjustment to 2.5 × 10^8^ platelets/mL; and the agonists 5 µg/mL Horm collagen (Helena Laboratories), 10‐‐20 µmol/L adenosine diphosphate (ADP; Sigma Aldrich Canada), 500 µmol/L murine thrombin receptor‐activating peptide (TRAP; AYPGKF‐NH_2_), and 0.5‐‐1.0 U/mL human thrombin (GFP only).

Shear‐induced platelet aggregation (SIPA) was evaluated using a HAAKE Mars rheometer (Thermo Electron Corporation) fitted with a C35/0.5° Ti (35 mm diameter) cone. Shear stress τ and shear rate γ were calculated automatically by HAAKE RheoWin 3 software. Whole blood from *Mmrn1^−/−^* and wild‐type mice was collected in 1:10 (v/v) 3.2% sodium citrate, and supplemented with 5 mmol/L CaCl_2_ and 93 µmol/L PPACK (to promote aggregation and prevent clotting, respectively) immediately before loading samples. Blood was sheared by the rotating cone in a 0.3 mm gap for 1 minute at room temperature before fixation with 1:4 (v/v) 0.625% paraformaldehyde (0.5% final). Fixed platelets were labelled in the dark using 1:8.3 (v/v) of anti‐mouse GpIbα (CD42b, Emfret Analytics) for 15 minutes before diluting samples 1:200 (v/v) in phosphate buffered saline and adding BD™ Liquid Counting Beads (165 beads/mL, Becton, Dickinson and Company, BD Biosciences). After mixing by gentle pipetting, 50 000 events were collected with a Beckman Coulter^®^ Epics^®^ XL‐MCL™ flow cytometer and Expo32™ software (Beckman Coulter). Events were gated for single platelets (defined by forward and side scatter characteristics) using FlowJo™ 10 (Becton, Dickinson and Company). Platelet concentration was estimated by comparing platelet and counting bead events to determine the % reduction in platelet concentration for sheared relative to unsheared samples.

### 2.9 Peptide synthesis

Triple‐helical Collagen Toolkit II and III peptides (full sequences reported elsewhere) and derivatives (sequences in Table S1 in supporting information) were generated as described,^29^ along with: CRP (GPVI ligand to activate platelets),^30^, ^31^ GFOGER (high‐affinity ligand for platelet integrin α_2_β_1_),^32^ Toolkit peptide III‐23 (containing the VWF ligand GPRGQOGVMGFO),^33^ and GPP (negative control).

### 2.10 Static platelet adhesion assays

Static platelet adhesion was evaluated largely as described,^34^ using *Mmrn1^−/−^* and wild‐type samples and wells pre‐coated with: 1 µg/well rMMRN1, murine Fg (±pre‐treatment with 0.2 U/mL thrombin), murine Fn, Horm collagen or collagen peptides (0.5 µg/well of each of two peptides or 0.33 µg/well of each of three peptides), or a range of peptide concentrations for titrations, before blocking and adding washed platelets (prepared as described,^34^ final: human: 1.6 × 10^8^ platelets/mL, 8 × 10^6^ platelets/well; mouse: 1.8 × 10^8^ platelets/mL, 9 × 10^6^ platelets/well). Unless otherwise specified, platelets were pre‐treated (without stirring) with 10 µg/mL of CRP (5 minutes, 37°C) immediately before testing.

### 2.11 Quantification of platelet adhesion and sizes of adherent platelet aggregates

Percent surface area covered by platelets was estimated using ImageJ (National Institutes of Health), and sizes of captured platelet aggregates on surfaces were estimated using representative regions (in‐focus) at the center of microcapillary channels, using the following objectives and areas: Horm collagen: 40× objective, ~60 mm^2^ square, 1020 × 1020 pixels; rVwf, fibrin(ogen), fibronectin, and collagen peptides: 20× objective, ~20 mm^2^ squares, 515 × 515 pixels. Grayscale images were converted to binary using the Threshold tool before separating features using the Watershed tool and counting using the Analyze Particles tool. As platelet aggregate sizes on Horm collagen varied greatly (~1‐‐40 000 µm^2^), data were binned and evaluated by frequency plots. Platelet aggregates captured onto rVwf, fibrin(ogen), fibronectin, and collagen peptides (sizes: ~1‐‐5000 µm^2^) were evaluated using mean feature sizes/image.

### 2.12 Statistical analyses

Two‐tailed independent or paired Student's *t*‐tests were used to evaluate data with normal distributions. Mann‐Whitney *U*‐tests were used to evaluate data with non‐normal distributions. One‐way or repeated measures analysis of variance were used to evaluate data with more than two groups (α < 0.05 considered significant). Bonferroni correction was used for post hoc multiple comparisons where *k* ≤ 6 or the Holm‐Sidak method where *k* > 6, with α < 0.05 considered significant for each family of comparisons. One‐tailed z test of population proportions was used to evaluate proportional data. Unless stated otherwise, data are reported as mean ± standard error of the mean (SEM).

## 3 RESULTS

### 3.1 Mice homozygous for Mmrn1 E3 deletion are viable and fertile, with normal platelet aggregation responses and a minor prolongation of bleeding time

**FIGURE 1:**
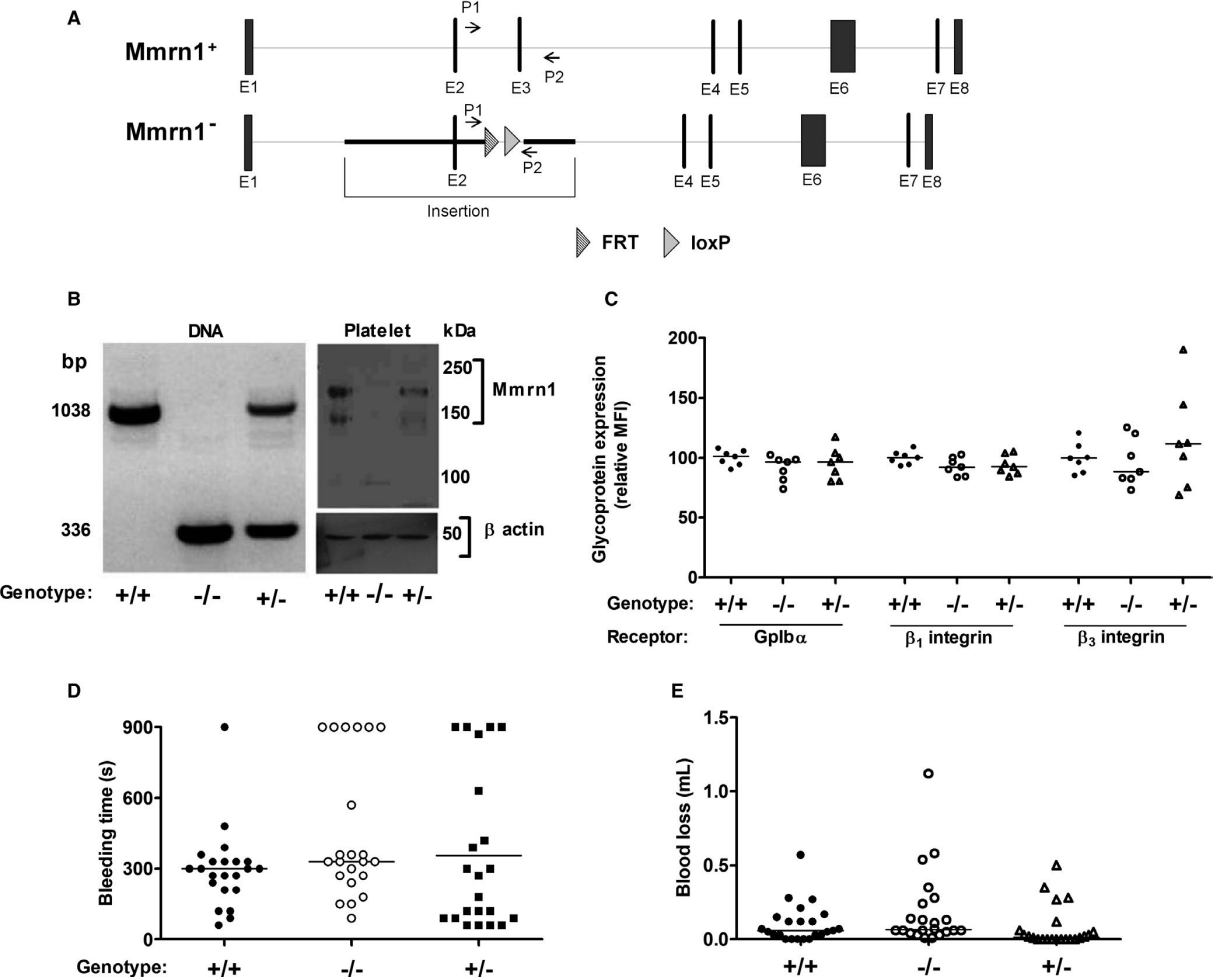
Evaluation of genotype, platelet phenotype, bleeding time, and wound blood loss of mice homozygous for multimerin 1 (*Mmrn1*) E3 deletion. A, Comparison of the *Mmrn1* gene in wild‐type (top) and *Mmrn1*‐deficient mice lacking E3 (bottom) with binding sites for primers P1 and P2 used for *Mmrn1* genotyping. The darker line indicates the region of *Mmrn1* replaced by the targeting vector via homologous recombination. B, Genotype (left) and phenotype (right) of *Mmrn1^+/+^*, *Mmrn1^+/−^*, and *Mmrn1^−/−^* mice analyzed by polymerase chain reaction and western blotting of platelet lysate using rabbit anti‐MMRN1, respectively. C, Flow cytometry quantification of platelet membrane GpIbα, β1 integrin, and β3 integrin (n = 7 per genotype). D, Bleeding time and (E) wound blood loss following distal tip tail transection (at a diameter of 1.5 mm), measured up to 900 s (n = 22 mice/group). Panels C–E compare data for *Mmrn1^+/+^* (solid circles), *Mmrn1^+/−^* (open triangles), and *Mmrn1^−/−^* (open circles) mice

Mice homozygous for the E3‐deleted *Mmrn1* allele (*Mmrn1^−/−^*; Figure 1A) were viable and fertile, and lacked detectable platelet Mmrn1 (Figure 1B), whereas *Mmrn1^+/−^* mice had reduced platelet Mmrn1 relative to wild‐type mice (Figure 1B). *Mmrn1^−/−^* and *Mmrn1^+/−^* mice had normal blood counts and Vwf levels (Table S2 in supporting information) and no overt bleeding or other phenotypic abnormalities. Additionally, compared to wild‐type platelets, *Mmrn1^−/−^* and *Mmrn1^+/−^* platelets showed comparable resting surface expression of glycoprotein (Gp)Ibα (CD42b) and β_1_ (CD29) and β_3_ integrins (CD61; Figure 1C), comparable thrombin‐induced platelet P‐selectin expression (CD62P; Figure S1A in supporting information) and only minor differences in activated α_IIb_β_3_ expression (*P* < .01; Figure S1B). BT (Figure 1D), the proportion of mice that did not stop bleeding by 900 seconds (6/22 versus 6/22 versus 1/22; *P* ≥ .09) and BT wound blood loss (Figure 1E) were not significantly different for wild‐type, *Mmrn1^+/−^*, and *Mmrn1^−/−^* mice even when the comparisons were extended to 22 mice per group (*P* ≥ .07).

Platelets from *Mmrn1^−/−^* mice showed: normal low shear LTA responses to 0.5‐‐1.0 U/mL thrombin (*P* ≥ .37; Figure S2A,B in supporting information), TRAP, collagen, and 10‐20 µmol/L ADP (*P* ≥ .13; Figure S2C‐F); normal WBA responses to collagen (*P* ≥ .49, Figure S3); and normal SIPA over a range of shear rates (5000‐15 000 s^−1^; *P* ≥ .49, Figure S4).

### 3.2 Mmrn1 loss impairs platelet adhesion, thrombus formation, and vessel occlusion in vivo

**FIGURE 2:**
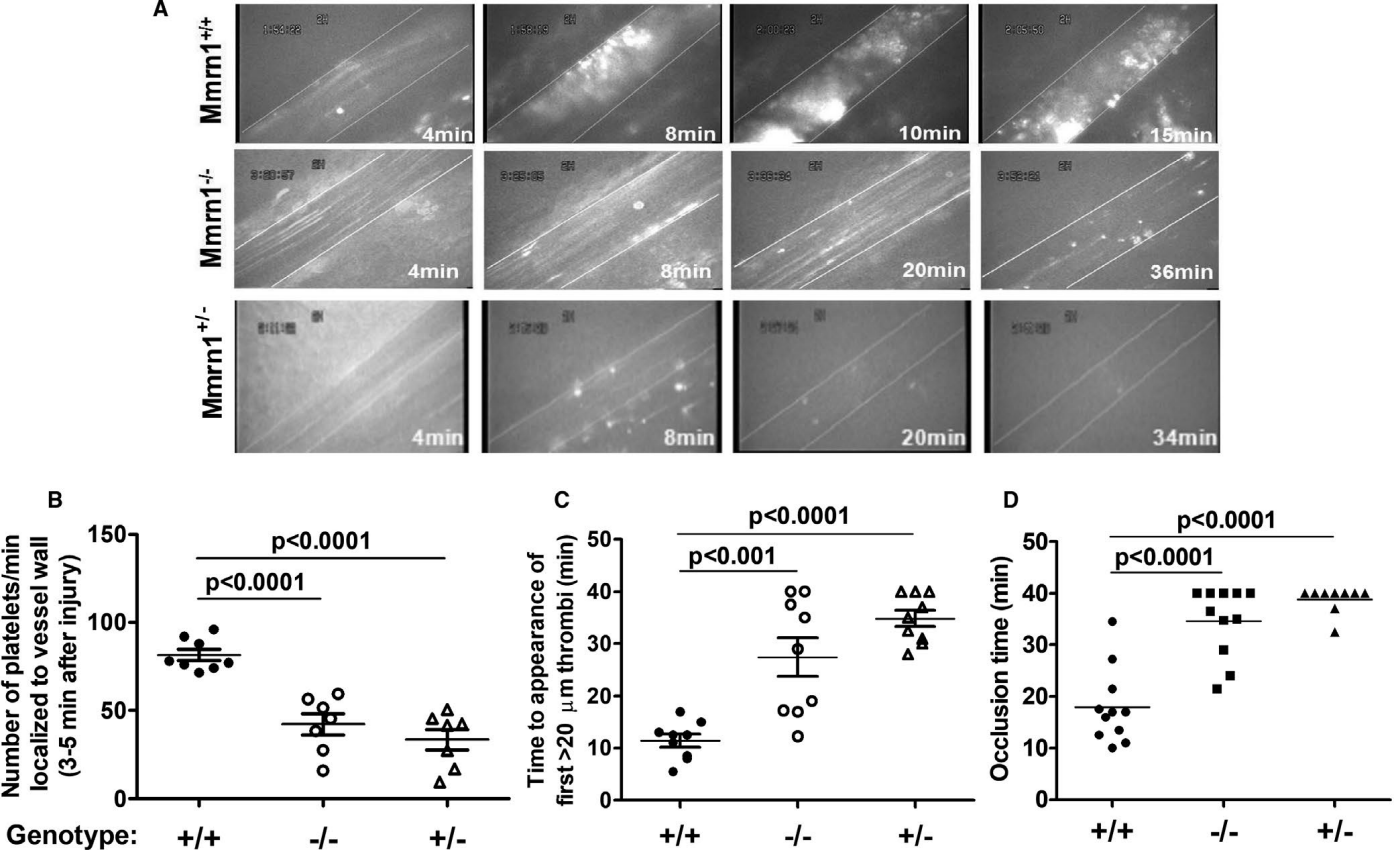
Thrombus formation in ferric chloride‐injured mesenteric vessels of mice with and without multimerin 1 (*Mmrn1*) E3 deletion. A, Representative images show calcein acetoxymethyl (AM) ester‐labeled platelet deposition and thrombus formation, captured using a Zeiss Axiovert 135 inverted epifluorescence microscope (Zeiss Oberkochen) and recorded on videotape (original magnification ×32; see representative Videos [Link], [Link], [Link] in supporting information) at different time points after ferric chloride injury, as indicated. B, Number of platelets localized to the vessel wall 3 to 5 minutes following exposure to ferric chloride (number of mice accessed/genotype: *Mmrn1^+/+^*: n = 8; others: n = 7). C, Time until the appearance of the first platelet‐rich thrombus ≥20 µmol/L in mice (n = 9 mice assessed/genotype). D, Time until total vessel occlusion (measured up to 40 minutes; number of mice assessed/genotype: *Mmrn1^+/−^*: n = 9; others: n = 11) mice. Symbols (panels B–D) indicate *Mmrn1^+/+^* (solid circle), *Mmrn1^−/−^* (open circle), and *Mmrn1^+/−^* (open triangle) mice

In the FeCl_3_‐induced mesenteric vessel injury model, both *Mmrn1^−/−^* and *Mmrn1^+/−^* mice showed impaired platelet localization between 3 and 5 minutes following injury (*P* < .001; Figure 2A,B), and delayed appearance of the first large thrombus (≥20 µm; *P* < .001; Figure 2C), compared to wild‐type mice, with 2/9 *Mmrn1^−/−^*, 3/7 *Mmrn1^+/−^* versus 0/9 *Mmrn1^+/+^* mice failing to form a thrombus ≥ 20 µm (*P* ≥ .067; Videos [Link], [Link], [Link] [in supporting information], respectively, show representative data for these mice). When large thrombi formed in arterioles of *Mmrn1^−/−^* or *Mmrn1^+/−^* mice, small platelet aggregates readily dissociated without re‐associating downstream. Although 11/11 *Mmrn1^+/+^* mice formed an occlusive thrombus by 10 to 35 minutes after FeCl_3_‐induced injury, 3/8 *Mmrn1^−/−^* mice and 7/7 *Mmrn1^+/−^* mice failed to form an occlusive thrombus by 40 minutes (*P* ≤ .014 for comparisons with *Mmrn1^+/+^* mice; *P* ≥ .29 for *Mmrn1^−/−^* to *Mmrn1^+/−^* mice comparisons; Figure 2D).

### 3.3 Mmrn1 loss selectively impairs platelet adhesion

**FIGURE 3:**
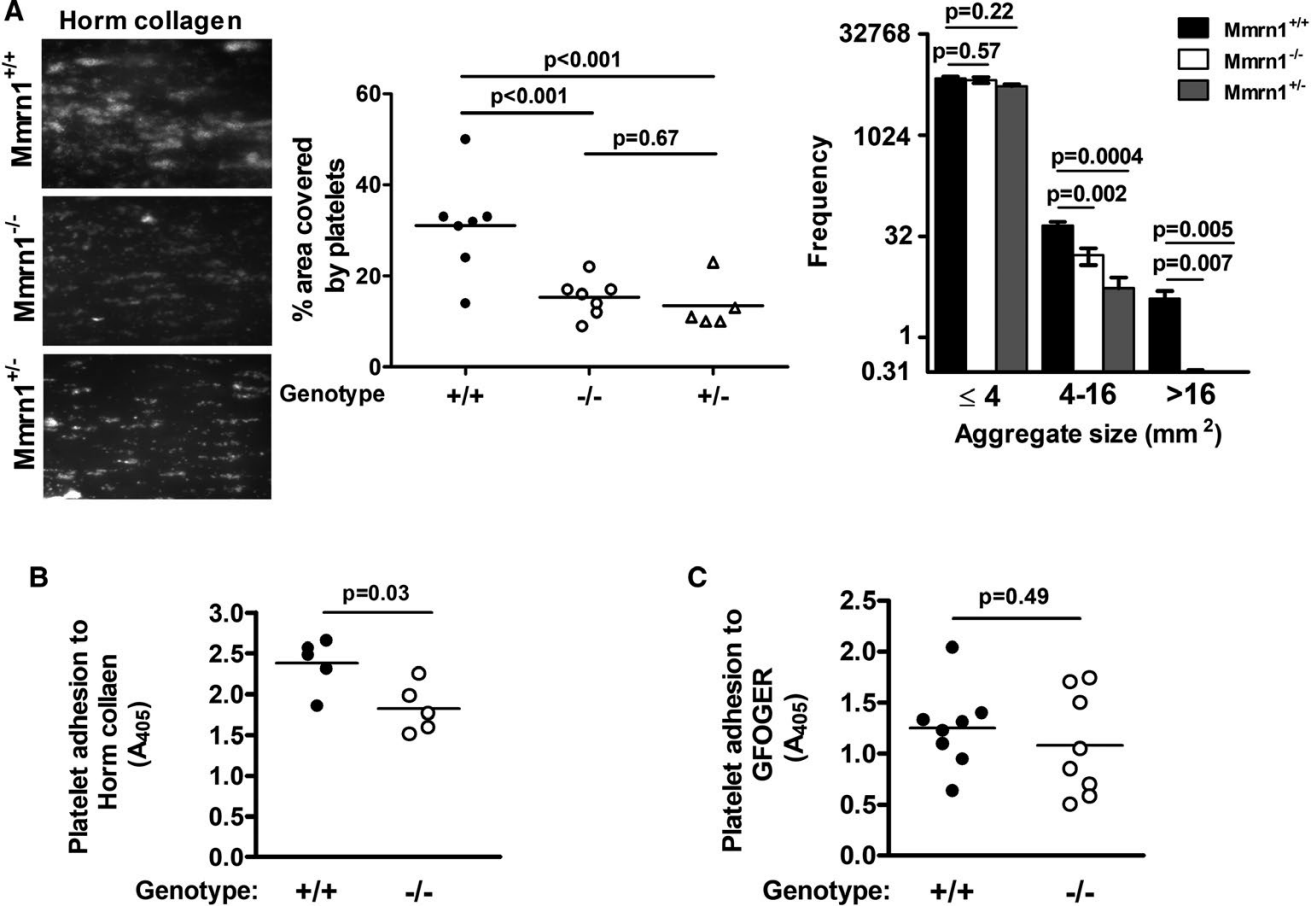
Effect of multimerin 1 (Mmrn1) deficiency on platelet adhesion to Horm collagen and GFOGER. A, Comparison of *Mmrn1^−/−^*, *Mmrn1^+/−^*, and wild‐type platelet adhesion to Horm collagen, tested at high shear (1500 s^−1^), assessed using representative images DiOC6(3)‐labeled platelets (left) and quantitative analyses of the surface area covered by platelets (middle) and the average frequency of different‐sized platelet aggregates captured onto collagen (right) (number of mice/genotype: *Mmrn1^+/−^*: n = 4, others: n = 6). Panels (B) and (C) show static adhesion of collagen‐related peptide‐activated platelets tested with: (B) Horm collagen (n = 5 mice/group) and (C) GFOGER (n = 8 mice/group; *Mmrn1^+/−^* mice not tested). Symbols indicate data for *Mmrn1^+/+^* (solid), *Mmrn1^+/−^* (open triangle), and *Mmrn1^−/−^* (open circle) mice

In high shear flow experiments (1500 s^−1^), with whole blood, both *Mmrn1^−/−^* and *Mmrn1^+/−^* platelets showed reduced adhesion to Horm collagen and failed to form the large adherent aggregates seen with wild‐type platelets (*P* ≤ .008, Figure 3A). Real‐time analyses of the findings for *Mmrn1^−/−^* and *Mmrn1^+/+^* samples (Videos S4 and S5 in supporting information, respectively), indicated that Mmrn1 deficiency was associated with less initial adhesion to collagen, and slower growth of platelet aggregates on collagen. Static platelet adhesion experiments verified that Mmrn1 deficiency impaired platelet adhesion to Horm collagen (*P* = .03; Figure 3B), without altering platelet adhesion to the triple‐helical collagen peptide GFOGER that functions as a high‐affinity α_2_β_1_ ligand (*P* = .49; Figure 3C). Mmrn1 deficiency also impaired platelet adhesion to Horm collagen in low shear flow (300 s^−1^) conditions (Figure S5 in supporting information).

**FIGURE 4:**
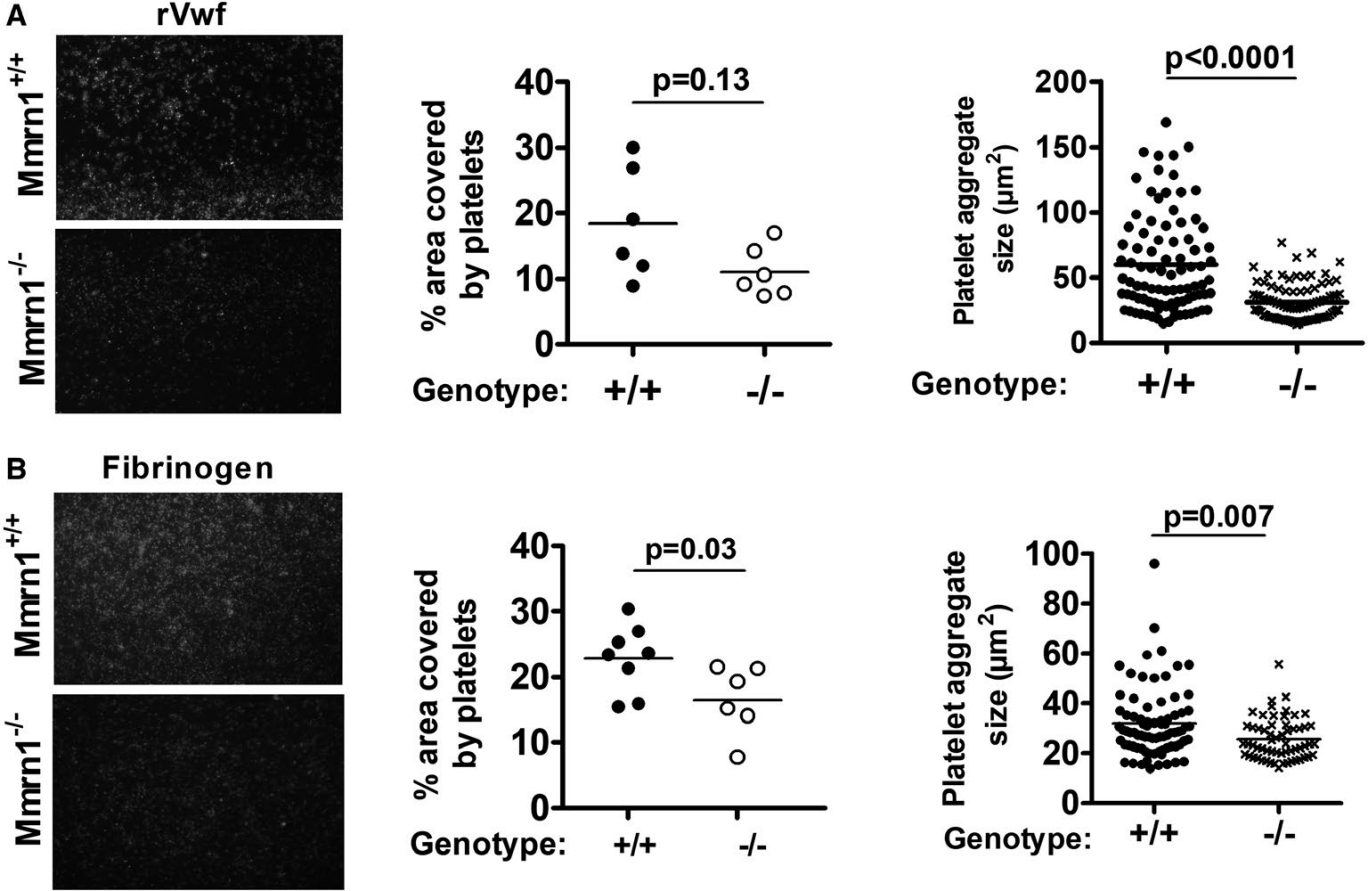
The effect of multimerin 1 (Mmrn1) deficiency on high shear (1500 s^−1^) platelet adhesion to recombinant Vwf and low shear (300 s^−1^) platelet adhesion to immobilized murine fibrinogen. Adhesion of wild‐type and *Mmrn1^−/−^* platelets was tested using whole blood and evaluated by representative images of adherent DiOC6(3)‐labeled platelets (left; captured by a Zeiss Axiovert 200 inverted epifluorescence microscope, coupled to a AxioCam MRc, and Axiovision software, Carl Zeiss Canada Ltd; original magnification ×20) with quantitative estimates of the surface area covered by adherent platelets (middle) and captured platelet aggregate sizes (right). A, Platelet adhesion to recombinant von Willebrand factor (n = 6 mice/genotype). B, Platelet adhesion to murine fibrinogen, evaluating using n = 8 *Mmrn1^+/+^* mice and n = 6 *Mmrn1^−/−^* mice. Symbols indicate data for *Mmrn1^+/^* ^+^ (solid bars and circles) and *Mmrn1^−/−^* mice (open bars and circles)

In high shear flow adhesion experiments (1500 s^−1^), *Mmrn1^−/−^* and *Mmrn1^+/+^* platelets showed similar adhesion to rVwf using whole blood (*P* = .13; Figure 4A) and washed platelets (*P* = .7; Figure S6 in supporting information), although *Mmrn1^−/−^* platelets were captured into smaller aggregates under these conditions (*P* < .0001).

Although rMMRN1 showed binding to human FG, fibrin, and FN (Figure S7A in supporting inforrmation; capitalized abbreviations indicate human proteins and mixed‐case abbreviations indicate mouse proteins), activated *Mmrn1^−/−^* and *Mmrn1^+/+^* platelets showed comparable static adhesion to these proteins (*P* ≥ .14, Figure S7B). The α_IIb_β_3_ blocking antibody 9D2 similarly reduced the adhesion of activated *Mmrn1^−/−^* and *Mmrn1^+/+^* platelets to rMMRN1 and other α_IIb_β_3_ ligands (Figure S7C). In low shear flow (300 s^−1^) adhesion assays with whole blood, activated *Mmrn1^−/−^* platelets showed a minor reduction in adhesion to Fg (*P* = .03), accompanied by a reduction in size of captured platelet aggregates (*P* = .007; Figure 4B), but normal adhesion to fibrin and Fn (Figure S8A‐D in supporting information).

### 3.4 MMRN1 binds to GPAGPOGPX motifs in fibrillar collagens, which enhance platelet adhesion and show high specificity for Mmrn1

**FIGURE 5:**
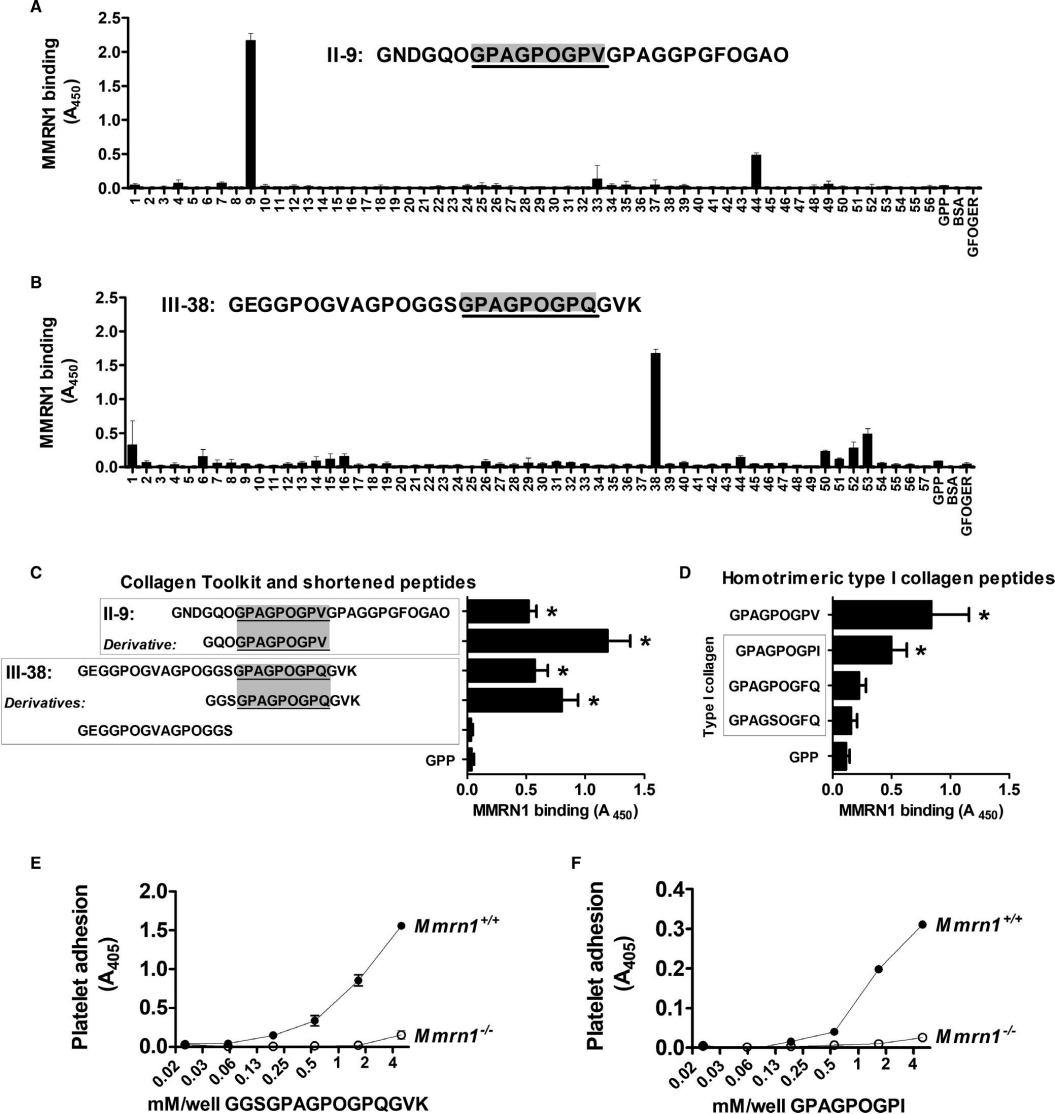
Multimerin 1(MMRN1) binding to collagen, evaluated using Collagen Toolkit and other triple‐helical peptides, and static platelet adhesion assays. Panels (A)–(B) show data from representative experiments to assess MMRN1 binding to Collagen Toolkit II (A) and Toolkit III (B) peptides, the control peptide GPP, and GFOGER. The sequence of the peptides showing the most binding is indicated in text. C, MMRN1 binding to Toolkit peptides II‐9 and III‐38 is compared to MMRN1 binding of shortened, derivatives of these peptides and GPP (negative control). D, MMRN1 binding to GPAGPOGPV and homotrimeric variants of the GPAGPOGPX sequence in collagen α1(I) and α2(I). E, Evaluation of the specificity of MMRN1‐binding peptide GGSGPAGPOGPQGVK by static platelet adhesion assays using collagen‐related peptide‐activated *Mmrn1^+/+^* (average for n = 4 mice) or *Mmrn1^−/−^* (average for n = 3 mice) platelets. F, Similar static platelet adhesion assay evaluation of the MMRN1‐specificity of GPAGPOGPI (average for n = 3 mice/group). In panels (A)–(C), absorbance values are corrected for non‐specific binding to bovine serum albumin (BSA). Bars and whiskers in Panels (C) and (D) represent the average and standard deviation of three identical experiments, each performed in triplicate. Symbols in Panels (E) and (F), respectively, show data for *Mmrn1* ^+/+^ (solid) and *Mmrn1^−/−^* (open) mice, and absorbance values are corrected for non‐specific adhesion by subtracting adhesion to GPP at each concentration tested

Collagen Peptide Toolkits were used to determine the locations and sequences in fibrillar vessel wall collagens that bind MMRN1. rMMRN1 bound two Collagen Toolkit peptides, II‐9 and III‐38, that share a GPAGPOGPX sequence where X is valine (II‐9) or glutamine (III‐38; Figure 5A,B). Tests with truncated derivatives indicated that MMRN1 bound to GPAGPOGPX but not to other regions of III‐38 (Figure 5C). In silico searches indicated that the GPAGPOGPX motif is unique to fibrillar collagens, and that the GPAGPOGPV locus (helix residues: 151‐‐159) of peptide II‐9 is poorly conserved in collagens I and III. Searches for motifs conserved with the MMRN1‐binding sequence in peptide III‐38 (GPAGPOGPQ, helix residues: 682‐‐690), and searches for variants of GPAGPOGPX elsewhere in collagen I (which consists of one α_2_ and two α_1_ chains) identified GPAGPOGPI at helix residues 667 to 675 in D‐period 3 of the α_1_(I) triple helix, and GPAGSOGFQ in D‐period 2 at residues 457 to 465 that aligns with a conserved GPAGPOGFQ sequence in α_2_(I). GPAGPOGPI in collagen I overlaps in sequence alignments with GPAGPOGPQ in collagen III, which has a 9‐residue extension at its N‐terminus, offsetting helix numbering. Testing of these collagen I sequences as homotrimeric triple‐helical peptides, in parallel with GPP (negative control) indicated that GPAGPOGPI (*P* = .0002), but not the variants GPAGPOGFQ (*P* = .12) or GPAGSOGFQ (*P* = .52), supported MMRN1 binding (Figure 5D).

As the GPAGPOGPX motifs that bind MMRN1 in human collagens I, II, and III are fully conserved in murine collagens, these motifs were further tested using peptide‐coated wells and mouse platelets. In contrast to CRP‐activated *Mmrn1^−/−^* platelets (which showed minimal adhesion), CRP‐activated *Mmrn1^+/+^* platelets showed dose‐dependent adhesion to GPAGPOGPQ and GPAGPOGPI (Figure 5E,F), indicating that GPAGPOGPX supports platelet adhesion by a Mmrn1‐dependent mechanism.

**FIGURE 6:**
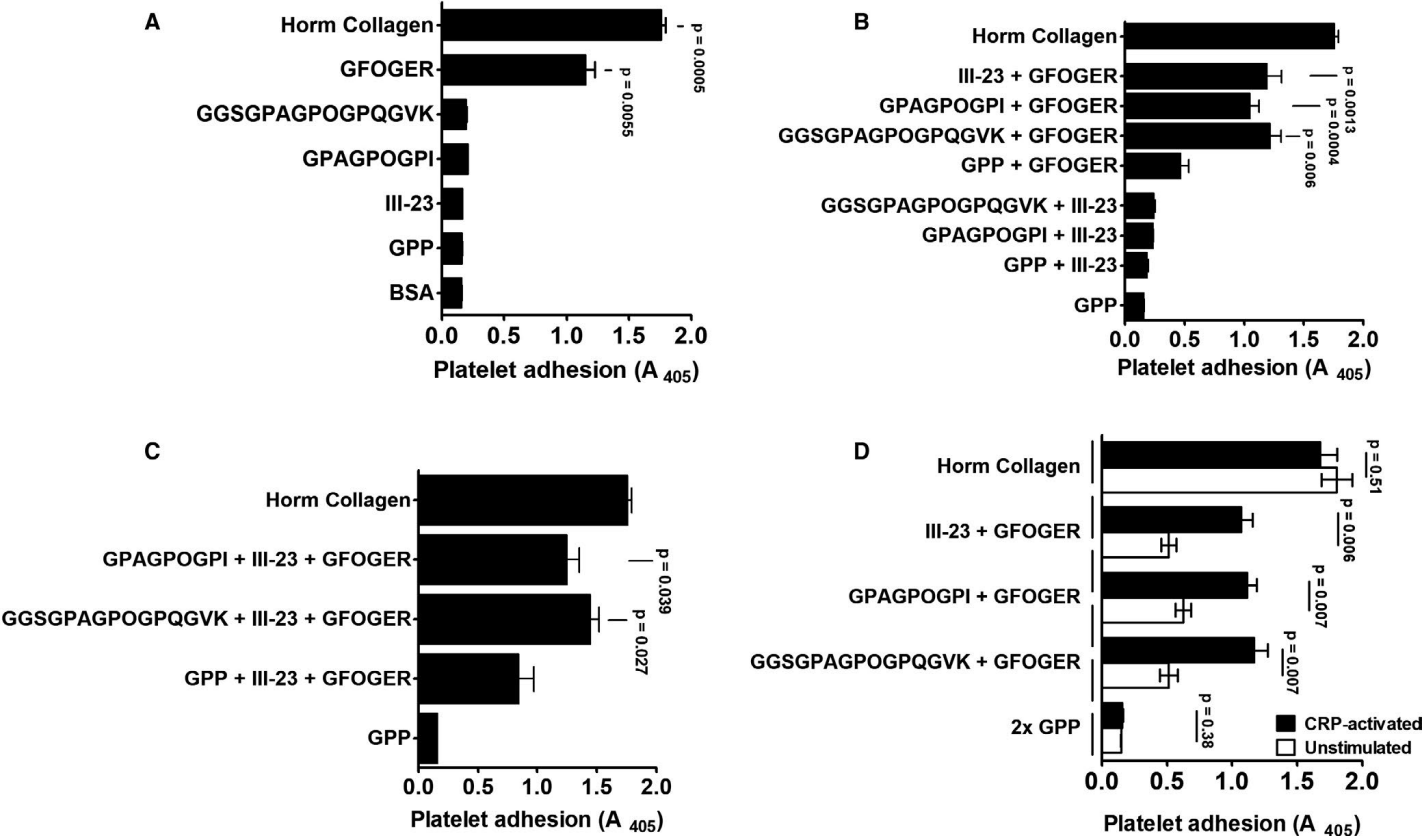
Synergistic effects of the multimerin 1 (MMRN1)‐binding GPAGPOGPX peptides and other collagen mimetic peptides on human platelet adhesion. Panels show adhesion of human platelets pre‐activated with 10 µg/mL collagen‐related peptide (CRP; to pre‐expose platelet MMRN1 and von Willebrand factor [VWF] and activate platelet integrins), unless otherwise indicated. Data are shown as mean absorbance ± standard error of the mean, for three different samples/group, each tested in triplicate to obtain a mean for each sample, evaluated with the indicated combination of peptides or with GPP (negative control) and Horm collagen (positive control). A, Platelet adhesion to all peptides except GFOGER was indistinguishable from background binding to GPP. B, The VWF‐binding sequence III‐23 and GPAGPOGPX peptides enhanced platelet adhesion to GFOGER but not to each other. C, Co‐presentation of III‐23 and GPAGPOGPX peptides increased platelet adhesion to GFOGER. D, Effect of platelet activation with 10 µg/mL CRP on platelet adhesion to GFOGER and GFOGER co‐presented with either GPAGPOGPX or III‐23

Although CRP‐activated human platelets did not significantly adhere to GPAGPOGPX peptides alone (Figure 6A), co‐presentation of GFOGER with GPAGPOGPX peptides, or with the VWF‐binding peptide III‐23, enhanced platelet adhesion (*P* ≤ .006; Figure 6B). When GFOGER was present, GPAGPOGPX peptides in combination with III‐23 further enhanced platelet adhesion (Figure 6B,C). The enhancing effect of GPAGPOGPX on platelet adhesion to GFOGER was increased by CRP activation to induce MMRN1 release (*P* ≤ .007, Figure 6D), either by adding CRP to the sample or to coating peptides (*P* > .24, Figure S9 in supporting information).

**FIGURE 7:**
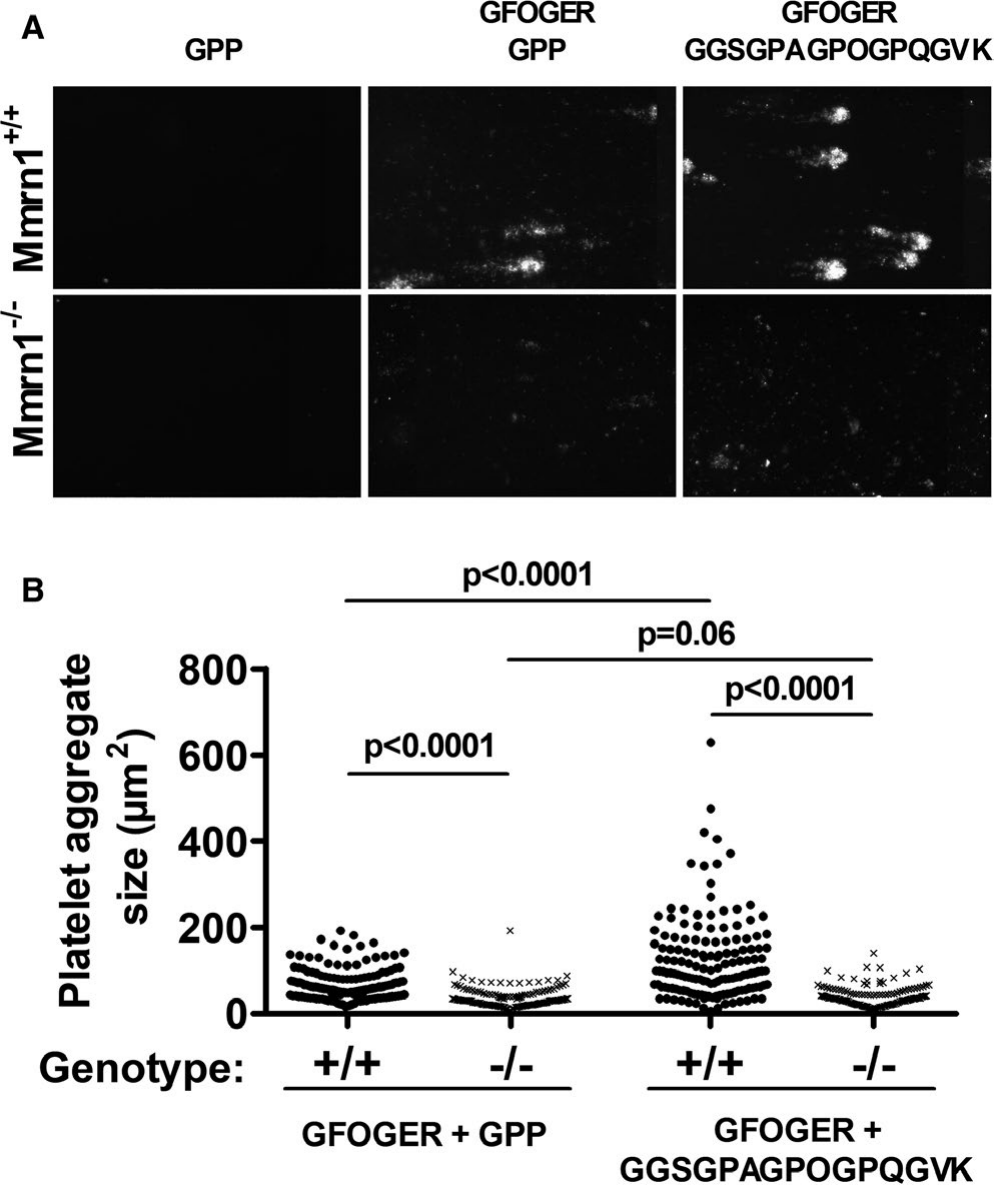
Adhesion of washed, collagen‐related peptide (CRP)‐activated wild‐type and multimerin 1 (Mmrn1)‐deficient mouse platelets to collagen peptides under high shear flow (1500 s^−1^). A, Representative images show endpoint adhesion of DiOC6(3)‐labeled CRP‐activated *Mmrn1^+/+^* and *Mmrn1^−/−^* platelets to immobilized triple‐helical collagen peptides, captured by a Zeiss Axiovert 200 inverted epifluorescence microscope coupled to a AxioCam MRc and Axiovision software (Carl Zeiss Canada Ltd.; original magnification ×20), and (B) mean platelet aggregate size on triple‐helical collagen peptides analyzed for each image captured (n = 5 mice/group, n = 15 images/experiment). Symbols in (B) represent *Mmrn1* genotype: +/+ (●) and *−/−* (x)

High shear flow experiments (1500 s^−1^) with CRP‐activated mouse platelets provided further evidence that GPAGPOGPX is highly specific for Mmrn1 as co‐presentation of GFOGER with GPAGPOGPQ increased the size of captured aggregates formed by wild‐type (*P* < .0001) but not *Mmrn1^−/−^* (*P* = .07) platelets (Figure 7A,B). *Mmrn1^−/−^* platelets were also noted to form smaller aggregates than wild‐type platelets on surfaces coated with GFOGER and GPP only (*P* < .001, Figure 7A,B), suggesting that Mmrn1 stabilizes platelet–platelet interactions through additional mechanisms.

## 4 DISCUSSION

Our goal was to evaluate platelet function in Mmrn1‐deficient mice and to identify motifs in collagen that support platelet adhesion by binding to MMRN1. Our main findings were: (a) partial and complete Mmrn1 loss impairs platelet adhesion and thrombus formation in vivo without causing a severe bleeding phenotype; (b) MMRN1 binds to FG, fibrin, and FN, but is not essential for platelet adhesion to these proteins, or to rVwf; (c) Mmrn1 loss preserves low shear and high shear platelet aggregation responses although it reduces platelet adhesion to fibrillar collagen and Fg, and the size of platelet aggregates captured onto these proteins; (d) partial and complete Mmrn1 impairs static, low shear, and high shear platelet adhesion to collagen; (e) Mmrn1 is not essential for platelet adhesion to rVwf or GFOGER but its loss reduces the size of platelet aggregates captured onto rVwf and GFOGER; and (f) fibrillar collagens that support platelet adhesion contain GPAGPOGPX motifs that are highly specific for Mmrn1 and work synergistically with other motifs to promote platelet adhesion.

Many studies of knockout mice have used the FeCl_3_‐induced mesenteric vessel injury model to assess platelet adhesion and thrombus formation. *α_2_^−/−^*,^35^ *FcRγ^−/−^* (which do not express GPVI),^36^ *Tsp1^−/−^*,^37^ *pFn^−/−^*,^38^, ^39^ *Fg^−/−^*,^40^ and *Vn^−/−^* mice ^41^ show impairments in one or two parameters of platelet adhesion and thrombus formation evaluated by this model. With *Mmrn1^−/−^* and *Mmrn1^+/−^* mice, platelet adhesion and thrombus formation were similarly impaired from start to finish in FeCl_3_‐injured vessels, reflected by reduced initial and final platelet adhesion and some mice failing to form any substantial thrombus. *Mmrn1^−/−^* and *Mmrn1^+/−^* mice did not exhibit spontaneous or fatal bleeding or significant alterations in BT. These data indicate that partial and complete Mmrn1 loss impairs platelet‐rich thrombus formation without incurring major impairments to hemostasis. This contrasts with the severely prolonged BT, reduced survival, or spontaneous bleeding reported for *Vwf^−/−^*,^42^ *Fg^−/−^*,^43^ *β_3_^−/−^*,^44^ and *GpIbα^−/−^* mice^45^—which also have impaired FeCl_3_‐induced thrombus formation. It is interesting that *Mmrn1^+/−^* mice, which had a milder platelet Mmrn1 deficiency than *Mmrn1^−/−^* mice, had impaired high shear collagen adhesion, and impairments in platelet adhesion and thrombus formation in the FeCl_3_ vessel injury model that were similar to, and not significantly different from, the impairments observed with *Mmrn1^−/−^* mice. These observations suggest that *Mmrn1* haploinsufficiency reduces Mmrn1 levels below what is needed to support Mmrn1‐dependent aspects of platelet function.

While we found that Mmrn1 is not required for platelet adhesion to Fg, fibrin, or Fn, its absence did reduce platelet adhesion, and the size of platelet aggregates captured onto Fg‐ but not fibrin‐coated surfaces under low shear flow. The latter discrepancies could reflect differences in the ultrastructure of surface‐adsorbed Fg versus fibrin, and/or differences in Fg‐ and fibrin‐α_IIb_β_3_ binding interactions.^46^, ^47^ It is also possible that the high‐affinity conformation of α_IIb_β_3_ or activation‐induced receptor clustering is required for platelets to bind Mmrn1, as activation enhances human platelet adhesion to MMRN1.^5^, ^6^ We suggest that Mmrn1 works synergistically with other proteins, including Fg and Vwf, to enhance platelet adhesion onto a growing platelet aggregate. The binding site(s) for MMRN1 on FG are unknown, but Mmrn1 does not appear to inhibit the ability of platelets to adhere to immobilized Fg, which occurs predominantly via the Fg γ‐chain binding to α_IIb_β_3_.^48^ Further, we did not detect an influence of Mmrn1 on platelet adhesion to immobilized Fn. Blockade of α_IIb_β_3_ partially inhibited static adhesion of *Mmrn1^−/−^* and wild‐type platelets to rMMRN1, similar to what we observed with human platelets,^5^ implicating the involvement of α_IIb_β_3_ and other, yet‐to‐be‐identified receptors in Mmrn1 binding.^5^

As platelets require activation for Mmrn1‐mediated adhesion, we suggest that Mmrn1 promotes adhesion after release from activated platelets and/or endothelial cells. The ligation of VWF to GPIbα induces activation of α_IIb_β_3_ and Ca^2+^ mobilization in platelets, but it is insufficient for α‐granule release,^49^ which could explain why we saw similar adhesion of *Mmrn1^−/−^* and *Mmrn1^+/+^* platelets to rVwf. There was likely some non‐intentional platelet activation in the experiments measuring platelet adhesion to rVwf at high shear given that *Mmrn1^−/−^* platelets were captured into smaller aggregates. Nonetheless, our findings suggest that MMRN1 participates in platelet adhesion only after platelets are tethered and have secreted granule contents. As platelets adherent to collagen are activated, with granule content secretion, Mmrn1 could be more important for platelet adhesion to collagen than to Vwf, as our data suggest. Larger MMRN1 multimers preferentially stay bound to platelets,^4^ which might enhance the avidity of Mmrn1 interactions with collagen fibers and Vwf multimers. As both *Mmrn1^−/−^* and *Mmrn1^+/−^* platelets showed impaired platelet adhesion to collagen in high shear flow, mild quantitative (eg, 50%) reductions in Mmrn1 appear to be sufficient to impair platelet–collagen interactions, which could have clinical implications. Additionally, Mmrn1 could bridge α_IIb_β_3_‐bound Fg on activated platelets to collagen, to further stabilize platelet adhesion. The idea that adhesive proteins bind one another to form large, macromolecular complexes has been proposed as a possible explanation for the supportive role of multimeric platelet VN,^41^ TSP‐1,^21^ and insoluble (crosslinked) plasma FN in platelet aggregate formation,^50^ and in platelet adhesion to collagen.^51^

**FIGURE 8:**
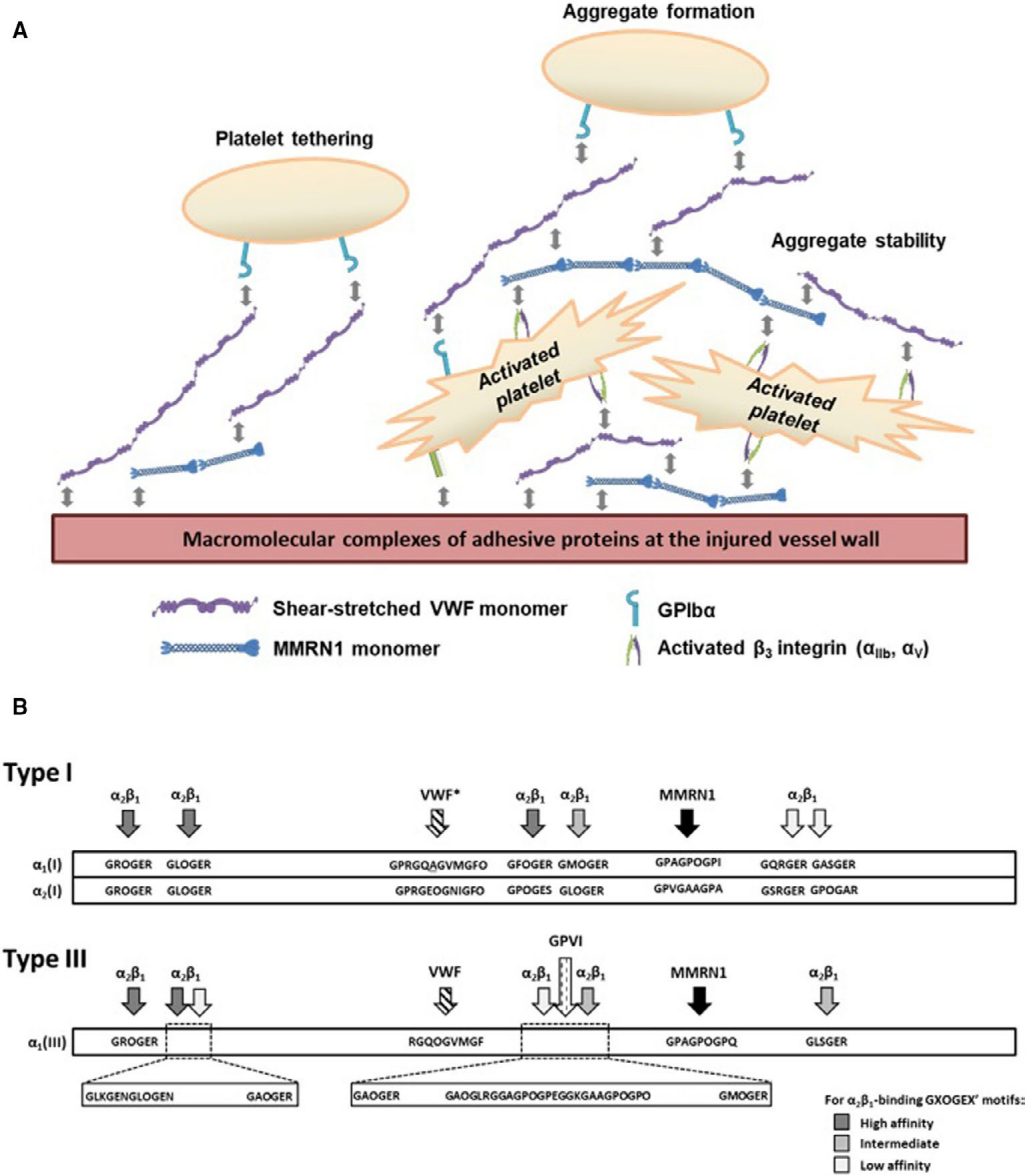
Proposed model of multimerin 1 (Mmrn1) functions in platelet adhesion and an updated model of the motifs in types I and III vessel wall fibrillar collagens that support platelet adhesion or activation. A, Proposed role of Mmrn1 in platelet adhesion. Following Mmrn1 release from platelets and endothelial cell storage granules, α_IIb_β_3_, α_v_β_3_, and other unidentified receptors mediate Mmrn1 binding to platelets. Mmrn1 binding to platelets increases the size and stability of platelet aggregates that are captured onto the macromolecular protein complexes that promote platelet–matrix and platelet–platelet interactions. Shear‐exposed and matrix bound von Willebrand factor (Vwf), fibrin(ogen), and fibronectin in these macromolecular protein complexes provide multiple binding sites for Mmrn1 attachment. Additionally, at sites of injury that expose blood to fibrillar collagen, Mmrn1 binds to GPAGPOGPX motifs to synergistically increase platelet adhesion to collagen beyond the adhesion supported by Vwf‐ and α_2_β_1_‐dependent mechanisms. B, Scale representation of the spatial arrangement of the functional sequences in the triple‐helical (COL) domain of human type I (top) and type III (bottom) collagen that support platelet adhesion or activation. *The α1‐α1‐α2 heterotrimer consisting of GPRGQAGVMGFO and GPRGEOGNIGFO in type I collagen has been verified to support VWF binding using heterotrimeric triple‐helical peptides.^72^ GXOGER sequences that bind α_2_β_1_, the VWF‐binding sequence GPRGQOGVMGFO, and the GPVI‐binding sequence GAOGLRGAGPOGPEGGKGAAGPOGPO have been previously described elsewhere

Our observations that complete and partial Mmrn1 loss impairs initial platelet adhesion and thrombus formation in the FeCl_3_ vessel injury model suggests Mmrn1 modifies macromolecular adhesive interactions in vivo. Potentially, Mmrn1 might bind to shear‐stretched Vwf multimers to form larger, heteropolymers that enhance platelet tethering and adhesion. As Mmrn1 loss reduced the size of platelet aggregates that adhered to collagen, rVwf, Fg, and GFOGER peptides, we suggest that Mmrn1 also functions to stabilize platelet–platelet interactions in wounds and injured vessels. Figure 8A summarizes the proposed role of Mmrn1 in platelet adhesion.

The GPAGPOGPX motif that binds MMRN1 is unique to collagens and distinct from other motifs identified with Collagen Toolkits (Figure 8B),^52^, ^53^, ^54^, ^55^, ^56^, ^57^, ^58^, ^59^, ^60^ including those that bind VWF,^33^ α_2_β_1_,^29^ and GPVI.^61^ While leukocyte‐associated Ig‐like receptor‐1 (LAIR‐1) binds to Collagen Toolkit peptide III‐38, LAIR‐1 binds to the repeated GPO motifs in peptide III‐38, not to the GPAGPOGPX sequence.^55^ The conserved MMRN1‐binding motif GPAGPOGPI lies at the same locus in the collagen α_1_(I) chain. The defective adhesion of Mmrn1‐deficient platelets to GPAGPOGPX motifs that were evident in static, low shear, and high shear flow conditions suggests that Mmrn1 supports platelet adhesion in wounds that expose blood to fibrillar collagen. Additionally, Mmrn1‐GPAGPOGPX binding could be one of the VWF‐independent mechanisms that mediates platelet adhesion and aggregate formation on collagen under low shear flow conditions.^62^

The in vivo defects in platelet adhesion and platelet‐rich thrombus formation of *Mmrn1^−/−^* and *Mmrn1^+/−^* mice may reflect other defects as collagen exposure in FeCl_3_ injured vessels appears minimal,^40^, ^63^ and the precise mechanisms that this model tests remain unclear.^64^, ^65^ While α_2_β_1_ and GpVI interact with fibrillar collagens to support platelet adhesion in vitro,^32^, ^61^, ^66^ impaired signalling, rather than impaired collagen interactions, is thought to underlie the impaired thrombus formation of *α_2_^−/−^* and *FcRγ^−/−^* (GpVI‐deficient) mice in FeCl_3_ injured vessels.^35^, ^36^ Mmrn1 loss could have other effects, given that MMRN1 binds factor V and can affect thrombin generation.^67^ In our study, we assessed the consequences of complete and partial Mmrn1 loss and in future studies, it would be interesting to test the contributions of platelet versus endothelial Mmrn1 in adhesion and thrombus formation in vivo, as both cells store Mmrn1 for activation‐induced secretion, and studies of other adhesive proteins stored in these cells (eg, von Willebrand factor, fibronectin) have provided insights on the relative contributions of the platelet versus endothelial stores.

Collagens are ubiquitous proteins that represent ~30% of total protein mass in mammals,^68^ with about 90% of collagens estimated to be type I fibrillar collagen.^69^ Injuries that sever or rupture a vessel would expose blood to the collagen‐rich outer layers of the vessel wall and extravascular connective tissues where Mmrn1 binding to GPAGPOGPX motifs may be very relevant to platelet adhesion. Disease‐causing mutations that impair MMRN1–collagen binding might affect platelet adhesion. While no pathogenic MMRN1 mutations are reported, G845R and G848R mutations in the GPAGPOGPI motif are associated with type II (perinatal lethal) osteogenesis imperfecta and a G852C mutation in GPAGPOGPQ is associated with type IV (vascular type) Ehlers‐Danlos syndrome, which causes hematomas, hemothorax, and vessel rupture.^70^, ^71^ Many mucocutaneous bleeding disorders remain uncharacterized and MMRN1 defects might go undetected by current bleeding disorder investigations. An assessment of platelet adhesion to collagen, and to combinations of CRP, GPAGPOGPX, and GFOGER peptides, could help identify cases of MMRN1 deficiency, but this would be a time‐ and resource‐intensive undertaking. Nonetheless, platelet MMRN1 deficiency might account for some platelet function abnormalities noted in gray platelet syndrome, Quebec platelet disorder, and αδ‐storage pool deficiency.

In summary, our study provides direct evidence that Mmrn1 contributes to platelet adhesion and thrombus formation in vivo, supports platelet adhesion and aggregate formation onto a variety of protein surfaces, and contributes to platelet adhesion to collagen through its interactions with Mmrn1‐specific GPAGPOGPX motifs in vessel wall fibrillar collagens.

## CONFLICTS OF INTEREST

RWF is Scientific Advisor and AB is the Lead Peptide Scientist at CambCol Laboratories Ltd. The other authors declare that they have no real or perceived conflicts of interest to disclose.

## AUTHOR CONTRIBUTIONS

A. Leatherdale and D. Parker designed and conducted experiments, analyzed and interpreted the findings, and led the manuscript writing, in collaboration with C. P. M. Hayward, who supervised the study. S. Tasneem designed and performed experiments with S. W. Hamaia and analyzed data. Y. Wang performed and analyzed the FeCl_3_ vessel injury experiments. D. Bihan and A. Bonna designed and synthesized collagen mimetic peptides. D. Lillicrap provided recombinant mouse Vwf, contributed to experimental design and interpretation, and edited the manuscript. P. L. Gross provided essential tools, guidance on microscopy and experimental design, and edited the manuscript. H. Ni provided essential tools and led the experimental design and supervision of the FeCl_3_ vessel injury experiments. B. W. Doble developed the strategy for generating Mmrn1‐deficient mice, which was carried out by S. Tasneem. R. W. Farndale guided the studies using Collagen Toolkit peptides to map MMRN1 binding motifs, supervised collagen peptide synthesis, and interpretation of peptide binding data, and edited the manuscript.

## Supporting information

Appendix S1

Video S1

Video S2

Video S3

Video S4

Video S5
