# Supplementary material for: Multimerin 1 supports platelet function in vivo and binds to specific GPAGPOGPX motifs in fibrillar collagens that enhance platelet adhesion
Source: J Thromb Haemost. 2020 Dec 17;19(2):547–61. doi: 10.1111/jth.15171 (PMC7898486; doi:10.1111/jth.15171)
Supplement: Supplementary file 1 — Appendix S1 [file JTH-19-547-s001.pdf]

Supplemental Methods

Generation of Mmrn1 deficient mice

The following strategy was used to generate selective Mmrn1-deficient mice: *Mmrn1*^{flxneo} mice (source: European Mutant Mouse Archive [EMMA], Neuherberg/Munich, Germany; EMMA ID:05337) were first crossed with Flp deleter mice (B6;J-Tg[ACTFLPe]9205Dym/J; Jackson Laboratories, Bar Harbor, ME, USA) to remove the Neo cassette and then crossed with transgenic global Cre deleter mice (B6.C-Tg[CMV-cre]1Cgn/J; Jackson Laboratories) to remove the floxed *Mmrn1* exon 3. The resultant mice were crossed with wild-type (*Mmrn1*$^{+/+}$) mice (C57BL/6J; Jackson Laboratories) to remove the *cre* and *flp* transgenes. Once the initial colony of *Mmrn1*$^{+/-}$ crosses was established, the breeding strategy was adapted to include crosses of wild-type and *Mmrn1*$^{-/-}$ breeding pairs, in addition to *Mmrn1*$^{+/-}$ crosses, to limit production of excess *Mmrn1*$^{+/-}$ mice. Every 3-5 generations, wild-type and *Mmrn1*$^{-/-}$ breeders were crossed back to generate new *Mmrn1*$^{+/-}$ mice for use as breeders and to establish new wild-type and null-null breeding pairs. Additionally, every 10 generations, *Mmrn1*$^{+/-}$ mice were crossed against wild-type C57BL/6J mice from Jackson Laboratories (Bar Harbor, ME). Experiments were performed using inbred *Mmrn1*$^{+/+}$, *Mmrn1*$^{+/-}$, and *Mmrn1*$^{-/-}$ obtained from these crosses.

Video 1. Platelet adhesion and thrombus formation in ferric chloride-injured mesenteric arterioles of *Mmrn1*$^{-/-}$ mice. Video was captured by a Zeiss Axiovert 135 inverted epifluorescence microscope using a 0.4NA x32 objective (Zeiss Oberkochen, Germany) and recorded on videotape.

Video 2. Platelet adhesion and thrombus formation in ferric chloride-injured mesenteric arterioles of *Mmrn1*$^{+/-}$ mice. Video was captured by a Zeiss Axiovert 135 inverted epifluorescence microscope using a 0.4NA x32 objective (Zeiss Oberkochen, Germany) and recorded on videotape.

Video 3. Platelet adhesion and thrombus formation in ferric chloride-injured mesenteric arterioles of wild-type mice. Video was captured by a Zeiss Axiovert 135 inverted epifluorescence microscope using a 0.4NA x32 objective (Zeiss Oberkochen, Germany) and recorded on videotape.

Video 4. Adhesion of *Mmrn1*$^{-/-}$ mouse platelets in whole blood to Horm collagen. Video was captured by a Zeiss Axiovert S 100 inverted epifluorescence microscope coupled to a VS4-1845 image intensifier assembly (Video Scope International Ltd., Sterling, VA, USA) and a Hamamatsu C9300 digital camera (Hamamatsu Corporation USA, Bridgewater, NJ, USA) using SlideBook 6 software (Intelligent Imaging Innovations, Denver, CO, USA) (original magnification x20). Time is shown to indicate images captured at the start of the video (30 sec.) and images capture at and after 4 min.

Video 5. Adhesion of wild-type mouse platelets in whole blood to Horm collagen. Video was captured by a Zeiss Axiovert S 100 inverted epifluorescence microscope coupled to a VS4-1845 image intensifier assembly (Video Scope International Ltd., Sterling, VA, USA) and a Hamamatsu C9300 digital camera (Hamamatsu Corporation USA, Bridgewater, NJ, USA) using SlideBook 6 software (Intelligent Imaging Innovations, Denver, CO, USA) (original magnification x20). Time is shown to indicate images captured at the start of the video (30 sec.) and images capture at and after 4 min.

	Amino acid sequence					
Peptide name	N-terminal Host	Guest sequence	C-terminal Host	Ligand	Function	Location
II-9*	GPC(GPP)$_5$	GNDGQOGPAGPOGPVGPAGGOGFOGAO	(GPP)$_5$GPC-amide	-	-	α_1(II)
CC7	GPC(GPP)$_5$	GQOGPAGPOGPV	(GPP)$_5$GPC-amide	-	-	α_1(II)
III-38*	GPC(GPP)$_5$	GEGGPOGVAGPOGGSGPAGPOGPQGVK	(GPP)$_5$GPC-amide	-	-	α_1(III)
CC6	GPC(GPP)$_5$	GEGGPOGVAGPOGGS	(GPP)$_5$GPC-amide	-	-	α_1(III)
CC13	GPC(GPP)$_5$	GGSGPAGPOGPQGVK	(GPP)$_5$GPC-amide	-	-	α_1(III)
AB-24	GPC(GPP)$_5$	GPAGPOGFQ	(GPP)$_5$GPC-amide	-	-	α_2(I)
AB-25	GPC(GPP)$_5$	GPAGSOGFQ	(GPP)$_5$GPC-amide	-	-	α_1(I)
AB-30	GPC(GPP)$_5$	GPAGPOGPI	(GPP)$_5$GPC-amide	-	-	α_1(I)
III-23*	GPC(GPP)$_5$	GPOGPSGPRGQOGVMGFOGPKGNDGAO	(GPP)$_5$GPC-amide	VWF	VWF binding	α_1(III)
GFOGER*	GPC(GPP)$_5$	GFOGER	(GPP)$_5$GPC-amide	Integrin $\alpha_2\beta_1$	Platelet adhesion	α_1(I)
CRP-XL*	GCO	(GPO)$_{10}$	GCOG-amide	Platelet GPVI	Platelet activation	-
GPP*	GPC(GPP)$_5$	(GPP)$_{10}$	(GPP)$_5$GPC-amide	-	Inert	-

Table S1. Details of triple-helical Collagen Toolkit and other collagen mimetic peptides used in the study. *Indicates previously described triple-helical peptides.

	Mmrn1$^{+/+}$	*Mmrn1*$^{-/-}$	*Mmrn1*$^{+/-}$
Platelets	523 ± 73 x 10^6 cells/ml	617 ± 66 x 10^6 cells/ml	684 ± 79 x 10^6 cells/ml
Erythrocytes	7.8 ± 0.3 x 10^{12} cells/l	7.6 ± 0.2 x 10^{12} cells/l	8.7 ± 0.3 x 10^{12} cells/l
Leukocytes	7.0 ± 1.0 x 10^9 cells/l	5.6 ± 0.9 x 10^9 cells/l	4.4 ± 0.8 x 10^9 cells/l
Plasma Vwf (% NMP)	100 ± 7%	108 ± 6%	95 ± 8%
Platelet Vwf (% NMP)	100 ± 10%	103 ± 16%	106 ± 15%

Table S2. Complete blood cell counts and Vwf levels for *Mmrn1*$^{+/+}$, *Mmrn1*$^{-/-}$ and *Mmrn1*$^{+/-}$ mice. Complete blood cell counts were obtained for n=8 mice/group. Plasma and platelets Vwf levels were measured and expressed as a percentage of normal mouse pool (NMP). Numbers of mouse samples analyzed were: plasma Vwf: n=10 mice/group; platelet Vwf; n=7 *Mmrn1*$^{+/+}$ mice and n=6 for the other groups of mice).

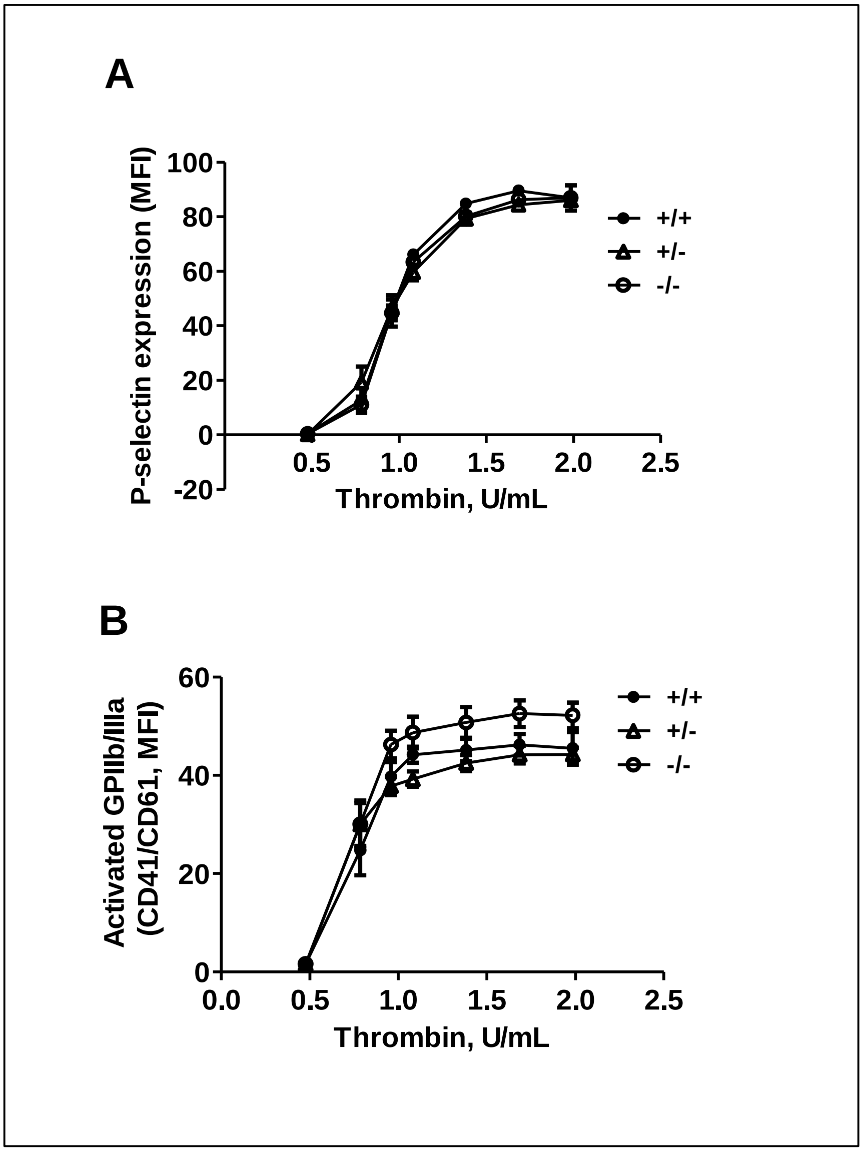

Figure S1. Flow cytometry analysis of activation-induced changes to surface glycoproteins on wild-type, *Mmrn1*$^{-/-}$, and *Mmrn1*$^{+/-}$ platelets. Panels show data for activated platelets (A, B) that were pretreated with different amounts of thrombin (n=7 mice/group for all analyses). Wild-type, *Mmrn1*$^{-/-}$, and *Mmrn1*$^{+/-}$mice had comparable activation-induced P-selectin expression. A small increase (relative to wild-type) was detected in the expression of activated $\alpha_{IIb}\beta_3$ on the surface of thrombin-activated *Mmrn1*$^{-/-}$ platelets only (p <0.01).

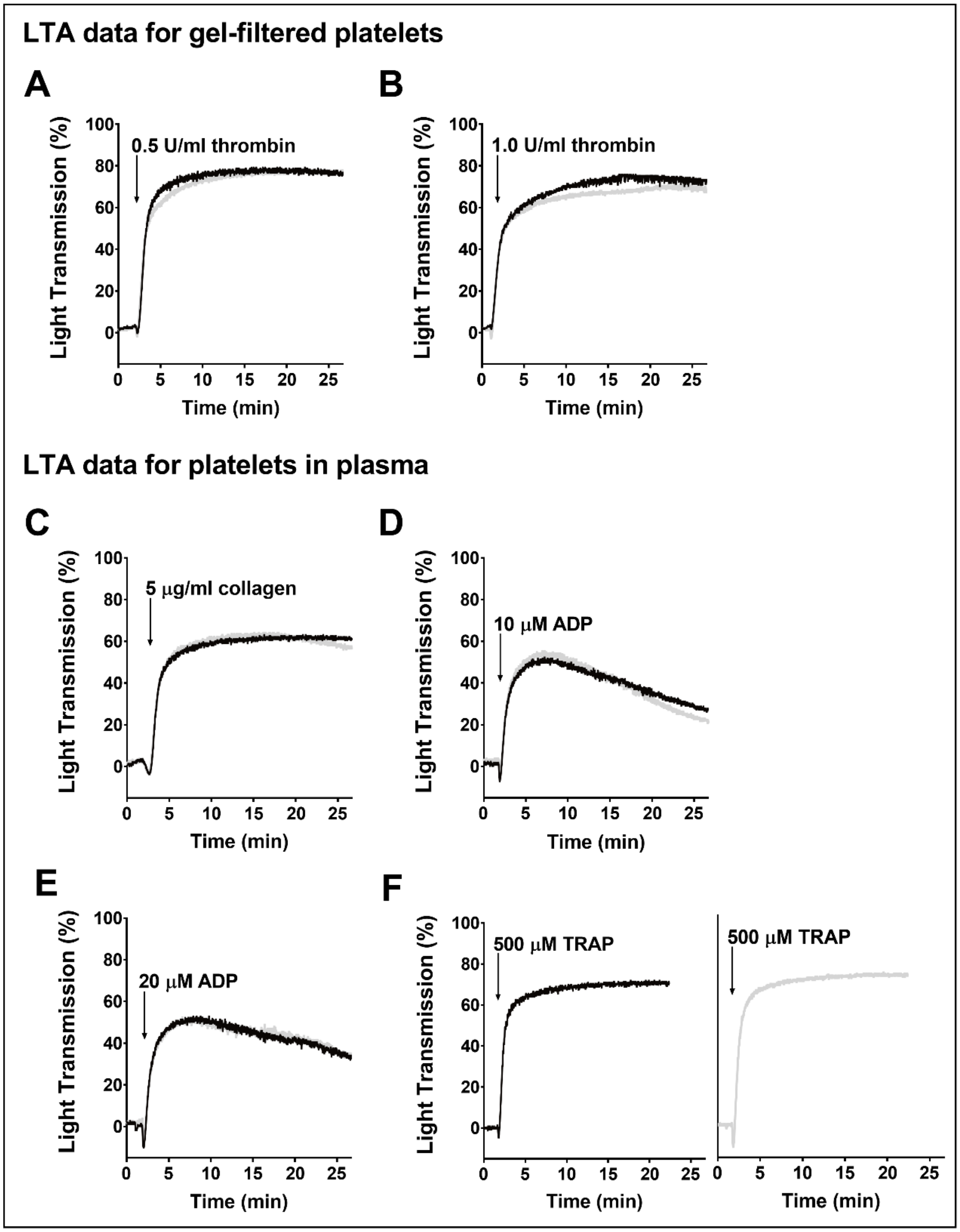

Figure S2. Comparison of the LTA responses of wild-type and Mmrn1-deficient mouse platelets to ADP, collagen, and TRAP. Panels show representative tracings for GFP (A,B) and PRP (C-F) samples stimulated with: (A) 0.5 U/ml and (B) 1.0 U/ml thrombin; (C) 5 µg/ml Horm collagen; (D) 10 and (E) 20 µM ADP; and (F) 500 µM TRAP. In panel F, curves are shown separately due to overlap. Black and gray indicate wild-type and *Mrmn1*$^{-/-}$ platelets, respectively.

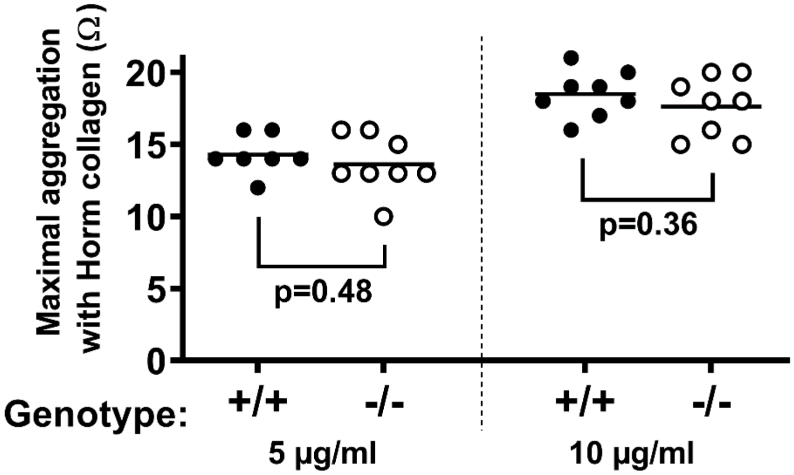

Figure S3. Whole blood aggregation responses of wild-type versus *Mmrn1*$^{-/-}$ mouse platelets tested with Horm collagen. Maximal aggregation responses were measured by electrical impedance (Ω) (*n*=7-8 mice/genotype). Symbols indicate Mmrn1 genotype (closed: *Mmrn1*$^{+/+}$; open: *Mmrn1*$^{-/-}$).

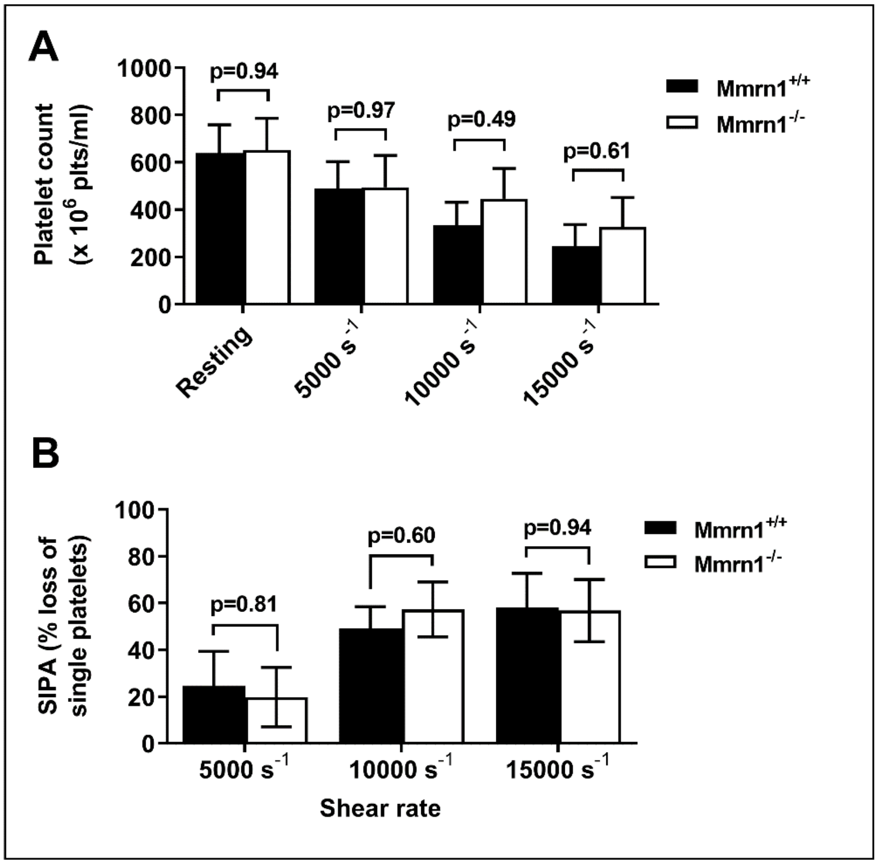

Figure S4. Shear-induced aggregation of wild-type versus *Mmrn1*$^{-/-}$ mouse platelets, tested in whole blood, in response to variable shear rates. Shear-induced aggregation of mouse platelets was quantified as changes to: (A) platelet counts and (B) % loss of single platelets in the whole blood samples. Bars and whiskers represent the mean and SEM, respectively (*n*=8 mice/group), and indicate *Mmrn1* genotype: solid: +/+; open: -/-.

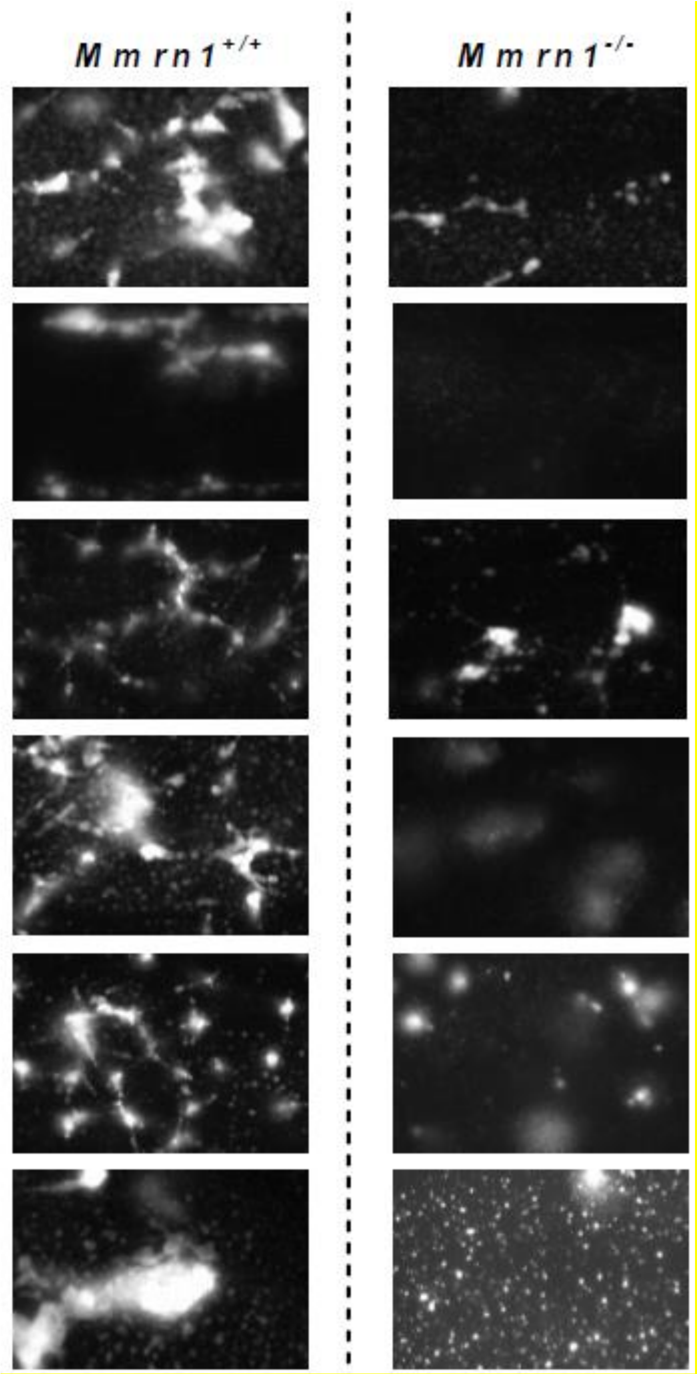

Figure S5. Adhesion of washed wild-type versus *Mmrn1*$^{-/-}$ mouse platelets to Horm collagen under low shear flow (300 s^{-1}). Single representative images from each biological replicate (n=6 mice/group) comparing the endpoint adhesion of DiOC6(3)-labeled *Mmrn1*$^{+/+}$ and *Mmrn1*$^{-/-}$ platelets to immobilized Horm collagen under low shear flow (300 s^{-1}). Given the heterogeneity of aggregate sizes and their appearance in multiple planes of focus, quantitative imaging was not possible under these conditions. Overall, the platelet aggregates captured onto Horm collagen at low shear were much less compact than those captured at high shear (see Fig. 3). Images were captured by a Zeiss Axiovert 200 inverted epifluorescence microscope coupled to a AxioCam

MRc and Axiovision software (Carl Zeiss Canada Ltd., Toronto, Canada) (original magnification x20).

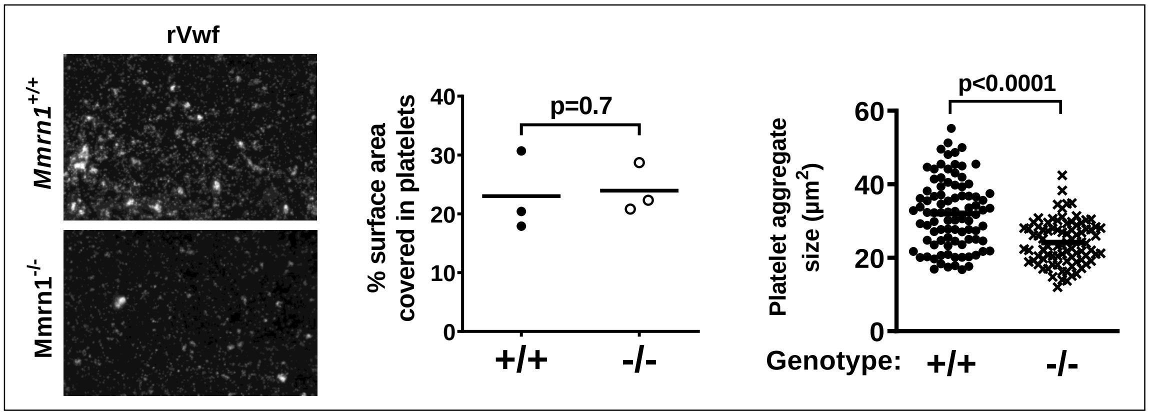

Figure S6. Adhesion of washed *Mmrn1*$^{-/-}$ and wild-type mouse platelets to recombinant Vwf under high shear flow (1500 s^{-1}). Representative images (left) showing adhesion of washed wild-type and *Mmrn1*$^{-/-}$ platelets, reconstituted with washed RBCs, as the surface area coverage (middle) and average sizes of platelet aggregates in each image (right) captured (n=3 mice/group, n=15 images/experiment). Images of adherent DiOC6(3)-labeled platelets were captured by a Zeiss Axiovert 200 inverted epifluorescence microscope, coupled to an AxioCam MRc, and Axiovision software (Carl Zeiss Canada Ltd., Toronto, Canada) (original magnification x20). Symbols indicate the *Mmrn1* genotype: +/+ (●) and -/- (o; x).

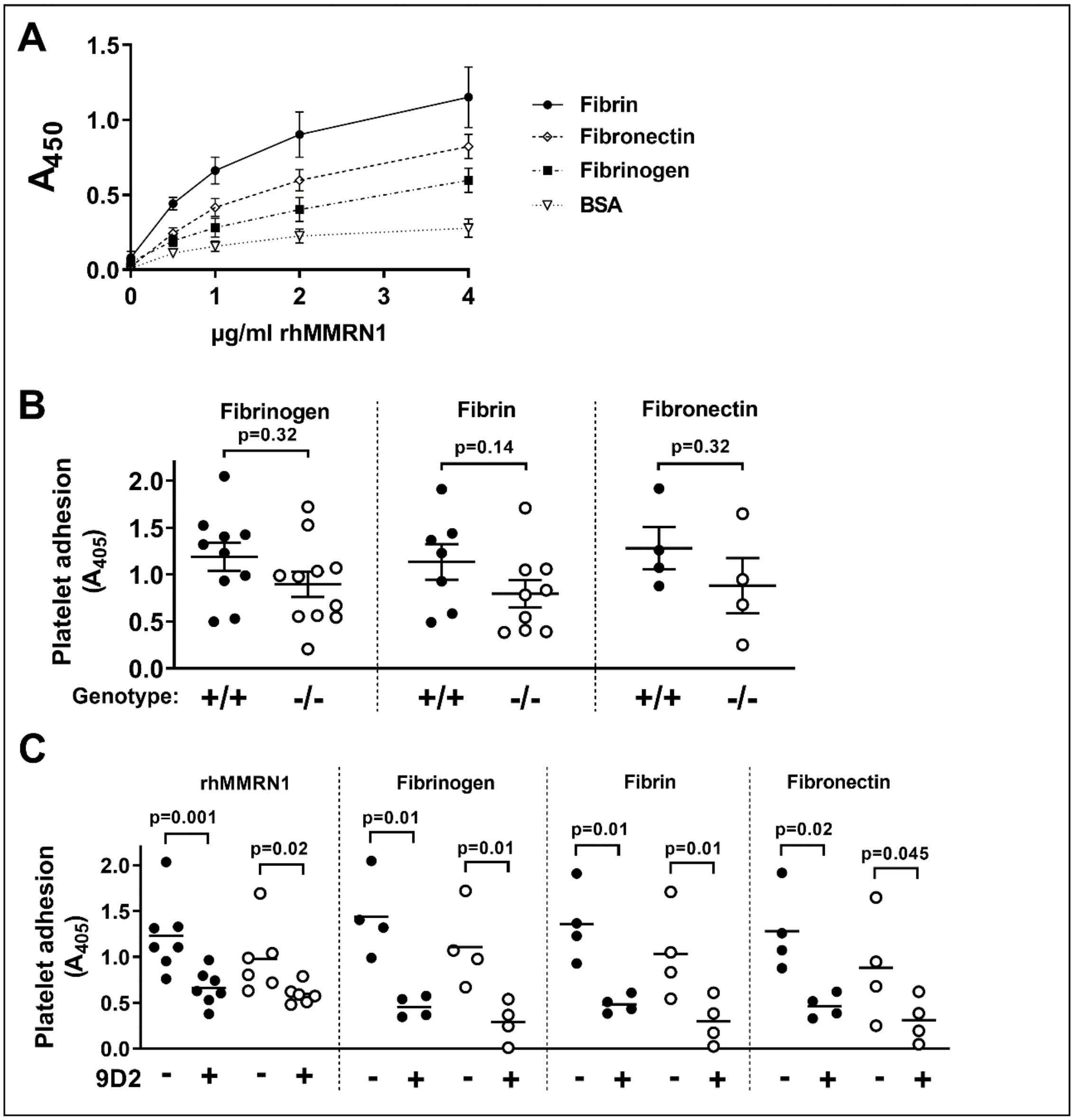

Figure S7. rhMMRN1 binding to human fibrinogen, human fibrin, or human fibronectin, static adhesion of washed, CRP-activated mouse platelets to murine fibrinogen, fibrin, or fibronectin, and the effect of anti-mouse β3 antibodies on static platelet adhesion to rhMMRN1 and other protein surfaces. (A) Absorbance values are representative data from a single experiment, where each determination was performed in triplicate to obtain a mean and SD. Data show the mean absorbance ± SD for each concentration of rhMMRN1 tested. (B) Static adhesion of washed wild-type and Mmrn1-deficient platelets pre-activated with 10 μg/ml CRP to pre-expose adhesive platelet α-granule proteins, including Mmrn1, and activate platelet integrins (Fg: n=10 *Mmrn1*$^{+/+}$, n=11 *Mmrn1*$^{-/-}$; fibrin: n=7 *Mmrn1*$^{+/+}$, n=9 *Mmrn1*$^{-/-}$; Fn: n=4 mice/group). (C) Static adhesion of washed, CRP-activated wild-type and Mmrn1-deficient platelets with or without anti-mouse β3 blocking antibody 9D2 (n=4 to 7 mice/group). Data are shown as mean absorbance measured in each sample, each tested in triplicate to obtain a mean. Symbols in panels B,C indicate the *Mmrn1* genotype (solid: +/+; open: -/-).

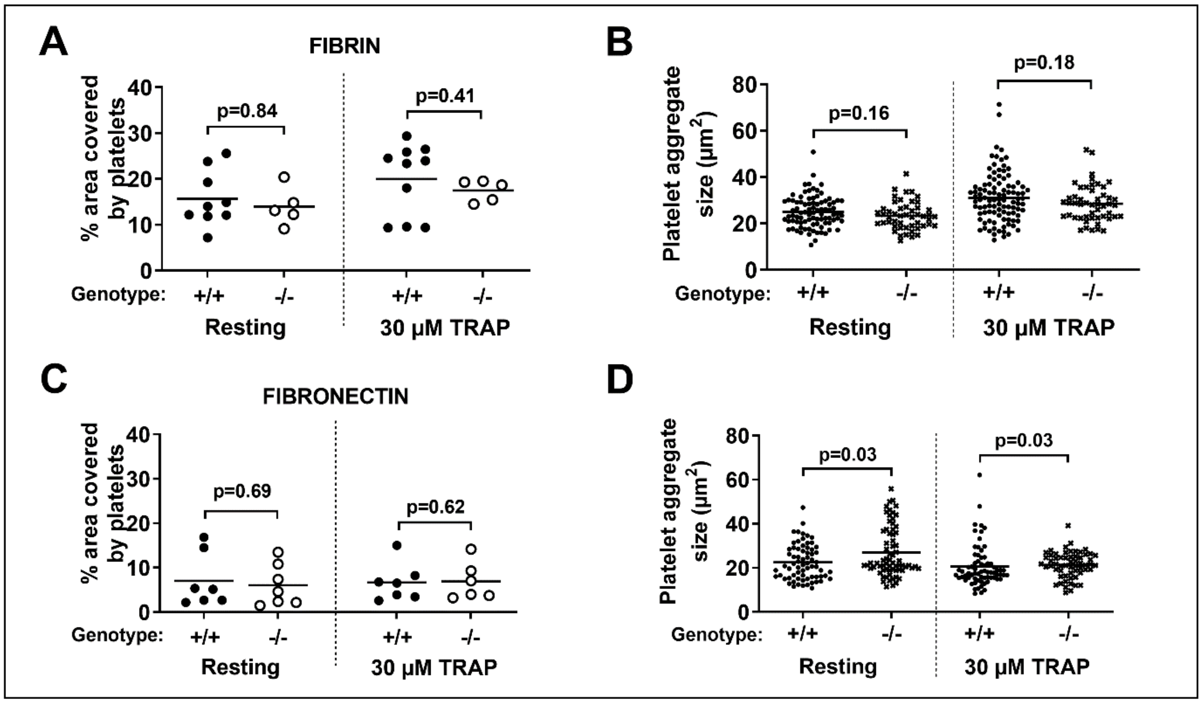

Figure S8. Effect of Mmrn1 loss on platelet adhesion to immobilized fibrinogen, fibrin, or fibronectin under low shear flow (300 s^{-1}). (A) Adhesion of resting or murine TRAP-activated wild-type and *Mmrn1*$^{-/-}$ platelets (tested in whole blood) to fibrin, shown as percent surface area coverage and (B) the average size of platelet aggregates in each image captured (resting *Mmrn1*$^{+/+}$: n = 9; TRAP-activated *Mmrn1*$^{+/+}$: n = 10; resting *Mmrn1*$^{-/-}$: n =5; TRAP-activated *Mmrn1*$^{-/-}$: n = 5). (C) Adhesion of resting or murine TRAP-activated wild-type and Mmrn1-deficient platelets (tested in whole blood) to fibronectin, shown as percent surface area coverage and (D) the average size of platelet aggregates in each image (right) captured (resting *Mmrn1*$^{+/+}$: n = 7; TRAP-activated *Mmrn1*$^{+/+}$: n = 7; resting *Mmrn1*$^{-/-}$: n =7; TRAP-activated *Mmrn1*$^{-/-}$: n = 6). Images of adherent DiOC6(3)-labeled platelets were captured by a Zeiss Axiovert 200 inverted epifluorescence microscope, coupled to a AxioCam MRc, and Axiovision software (Carl Zeiss Canada Ltd., Toronto, Canada) (original magnification x20). Symbols in panels A-D represent *Mmrn1* genotype: +/+ (●) and -/- (o; x).

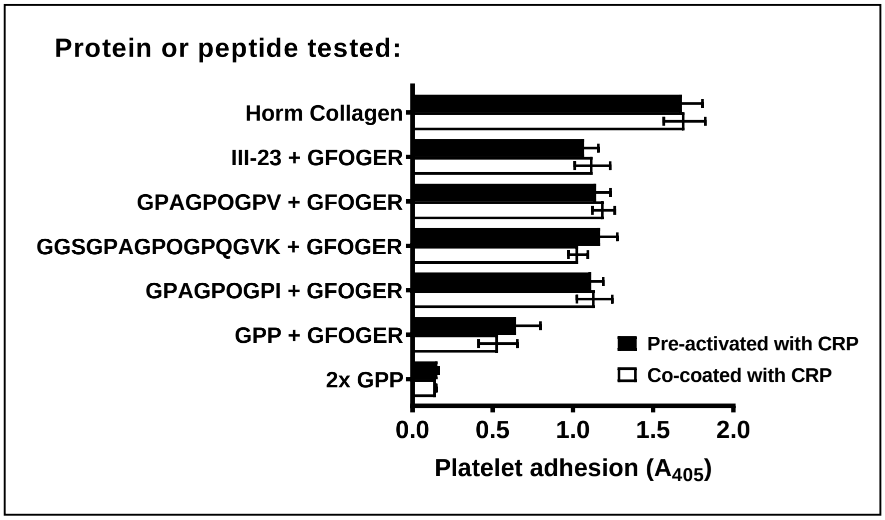

Figure S9. Comparison of the effects of CRP pre-activation or within-well CRP activation on static human platelet adhesion to triple-helical collagen peptides that bind MMRN1 or other ligands. Panel illustrates that there was comparable static platelet adhesion data (shown as mean absorbance ± SEM, for 3 different samples/group) for tests with CRP-pre-activated platelets and platelets activated by CRP in the peptide mixture used to coat the wells, as outlined in methods.
